# Self‐Assembled (Aza)BODIPY Dyes for Biomedical in Vivo Imaging

**DOI:** 10.1002/anie.202525491

**Published:** 2026-01-19

**Authors:** Antonia Albers, Max Masthoff, Gustavo Fernández

**Affiliations:** ^1^ Organisch‐Chemisches Institut Universität Münster Corrensstraße 36 48149 Münster Germany; ^2^ Klinik für Radiologie Universität und Universitätsklinikum Münster Albert‐Schweizer‐Campus 1 48149 Münster Germany

**Keywords:** (aza)BODIPY dyes, Amphiphilic systems, Contrast agents, Multimodal Imaging, Self‐assembly

## Abstract

4,4‐difluoro‐4‐bora‐3a,4a‐diaza‐s‐indacenes (BODIPYs) and their N‐containing analogs (aza‐BODIPYs) are highly versatile organic dyes that combine excellent photophysical, electronic, and supramolecular properties with high chemical stability. These features have enabled their use in diverse applications such as bioimaging, targeted drug delivery, and functional self‐assembled materials. Their broad structural modularity allows different stacking modes with controlled exciton coupling during self‐assembly, resulting in supramolecular materials with tunable spectroscopic and morphological properties. This is particularly relevant in aqueous media, where tunable near‐infrared (NIR) absorption and emission are essential for advanced contrast agents (CAs) in biomedical imaging. Besides *meso*‐heteroatom modification, J‐aggregation stands out as especially advantageous for achieving red‐shifted absorption and emission, thereby improving tissue penetration and reducing autofluorescence and light scattering. Of particular promise is the concept of pathway complexity, whereby a single (aza)BODIPY scaffold can access multiple aggregated states with distinct photophysical signatures, enabling stimuli‐responsive and multifunctional imaging behavior. Hence, understanding the interplay between (aza)BODIPY molecular design, exciton coupling in aqueous media and their resulting supramolecular and bioimaging properties is of utmost importance. This minireview discusses recent advances in (aza)BODIPY‐based self‐assembled structures used as CA platforms for in vivo biomedical imaging and highlights the structure–property relationships that govern their performance.

## Graphical abstract

This minireview classifies (aza)BODIPY dyes according to their molecular stacking modes and aggregate types (H‐, oblique‐, or J‐type) in aqueous media, correlating these features with their multimodal imaging performance. By linking molecular design, exciton coupling, and pathway‐dependent assembly, we provide a framework for engineering next‐generation (aza)BODIPY systems with tunable, multifunctional biomedical imaging capabilities.

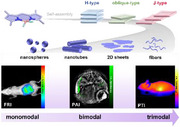

## 1 Introduction

4,4‐difluoro‐4‐bora‐3a,4a‐diaza‐s‐indacenes (BODIPYs) and their N‐containing analogs (aza‐BODIPYs) have attracted growing interest over the past few decades because of their exceptional electronic and photophysical characteristics,^[^ ^1^, ^2^, ^3^ ^]^ including high photo‐ and chemical stability, tuneable (NIR) absorption and emission, and high fluorescence quantum yields.^[^ ^1^ ^]^ Such properties make (aza)BODIPYs highly versatile, enabling applications across diverse areas including light‐harvesting,^[^ ^4^ ^]^ chemical sensing,^[^ ^5^ ^]^ targeted drug delivery,^[^ ^6^ ^]^ and more recently biomedical contrast agents (CAs).^[^ ^7^ ^]^ In this regard, biomedical imaging enables the visualization of biological processes at the molecular level and hence, has become indispensable for research and tomorrow's personalized medicine by supporting non‐invasive diagnosis and real‐time monitoring of disease.^[^ ^8^, ^9^ ^]^ Among the various diagnostic modalities, fluorescence imaging (FI), photoacoustic imaging (PAI), and photothermal imaging (PTI) critically rely on the availability of CAs with tailored optical properties. A key requirement for such agents is efficient operation within the NIR biological window (700–1700 nm), where reduced light scattering, minimized absorption of biomolecules, and suppressed tissue autofluorescence allow deeper tissue penetration and enhanced signal‐to‐noise ratios. Consequently, (NIR) fluorescence imaging has emerged as a particularly powerful approach, combining high sensitivity with the ability to probe tissue non‐invasively and at significant depth.^[^ ^10^ ^]^ Conventional BODIPY dyes exhibit outstanding photophysical properties; however, their absorption maxima rarely exceed 700 nm, which limits their application for NIR bioimaging. To expand their utility, various structural modification strategies have been developed to red‐shift their absorption, e.g. extending the *π*‐system by aryl‐ or styryl‐substitution of the BODIPY core.^[^ ^11^, ^12^ ^]^ Another effective approach is the introduction of a *meso*‐heteroatom, as realized in aza‐BODIPYs, which shifts the absorption into the NIR region.^[^ ^2^, ^13^ ^]^ The red‐shift arises primarily from the electronegative nature of the nitrogen atom, which lowers the LUMO energy while leaving the HOMO nearly unaffected, thereby reducing the HOMO–LUMO energy gap. In addition, the aryl substituents can electronically interact with the *π*‐core, extending the delocalization of the frontier orbitals. This further reduces the HOMO–LUMO gap and enhances the red‐shift of the absorption.^[^ ^14^ ^]^ Particularly, the (aza)BODIPY scaffold offers a versatile platform for further functionalization: its multiple reactive sites can be substituted with electron‐donating or electron‐withdrawing groups, creating electronic push–pull effects that can tune the absorption profiles.^[^ ^15^, ^16^ ^]^

While these molecular engineering strategies have proven effective in modulating the optical properties of (aza)BODIPYs, their practical translation into biological applications poses another major challenge; most organic fluorescent probes are inherently hydrophobic, which significantly limits their solubility in aqueous media. To address this issue, the dyes are often encapsulated or chemically conjugated to polymers to enhance their biocompatibility and broaden their applicability.^[^ ^17^, ^18^, ^19^ ^]^ Depending on the nature of the carrier (e.g., DSPE‐PEG, Pluronic F127, POEGMA23–PAsp20) and the local dye concentration, nanoencapsulation can preserve the monomeric state of hydrophobic chromophores or promote dye–dye interactions that lead to distinct excitonic coupling. Such encapsulation can influence aggregation behavior and photophysical properties through several mechanisms: 1) increased local concentration can promote aggregate formation; 2) the carrier environment—including polarity, hydrophobicity, and rigidity—can bias aggregate geometry and packing; 3) encapsulation can suppress aggregation‐caused quenching (ACQ) or stabilize pre‐formed aggregates depending on dye loading and carrier properties; and 4) the carrier matrix can template specific aggregate arrangements, further modulating the photophysical behavior compared to free‐dye aggregates.^[^ ^20^, ^21^ ^]^ Although polymer‐based carrier systems have shown great promise, further improvements are needed to address issues such as loading efficiency, fabrication complexity, and reproducibility, properties of utmost importance if clinical application is desired.^[^ ^22^ ^]^

Self‐assembly through rational molecular design represents a powerful complementary strategy to address these challenges,^[^ ^23^, ^24^, ^25^, ^26^, ^27^, ^28^ ^]^ enabling precise control over key parameters such as colloidal stability, photophysical characteristics, and aggregate size and shape, all of which are essential for effective biomedical imaging. This approach relies on the spontaneous organization of molecular building blocks into ordered supramolecular structures driven exclusively by non‐covalent interactions, such as aromatic, electrostatic, or hydrogen bonding interactions, without the need for external templates, fields, chemical additives, or carriers.^[^ ^29^, ^30^ ^]^ Owing to their excellent photophysical properties, (aza)BODIPY dyes have been widely employed to construct well‐defined supramolecular nanostructures with diverse molecular packing arrangements and morphologies, often incorporating functional moieties such as diagnostic agents, targeting ligands, or therapeutics.^[^ ^31^, ^32^, ^33^, ^34^, ^35^ ^]^ Beyond their optical performance, the physicochemical characteristics of these assemblies (e.g., size, charge, morphology) govern biodistribution and cellular uptake. By leveraging the enhanced permeability and retention (EPR) effect,^[^ ^8^ ^]^ nanoscale assemblies can selectively accumulate in tumors, serving as versatile platforms for tumor microenvironment (TME)–responsive theranostic CAs activated by stimuli such as pH, enzymes, hydrogen peroxide, or glutathione (GSH).^[^ ^36^, ^37^ ^]^ In addition to their diagnostic imaging capabilities, these CAs can also implement therapeutic strategies such as photodynamic (PDT) and photothermal therapy (PTT). In PDT, light‐activated photosensitizers generate reactive oxygen species (ROS) to induce localized cytotoxicity: Type I PDT involves electron transfer to produce radicals, while Type II PDT transfers energy to molecular oxygen to generate singlet oxygen. Alternatively, PTT utilizes photothermal agents that convert absorbed light into heat, enabling targeted hyperthermia and tissue ablation.

**Scheme 1:**
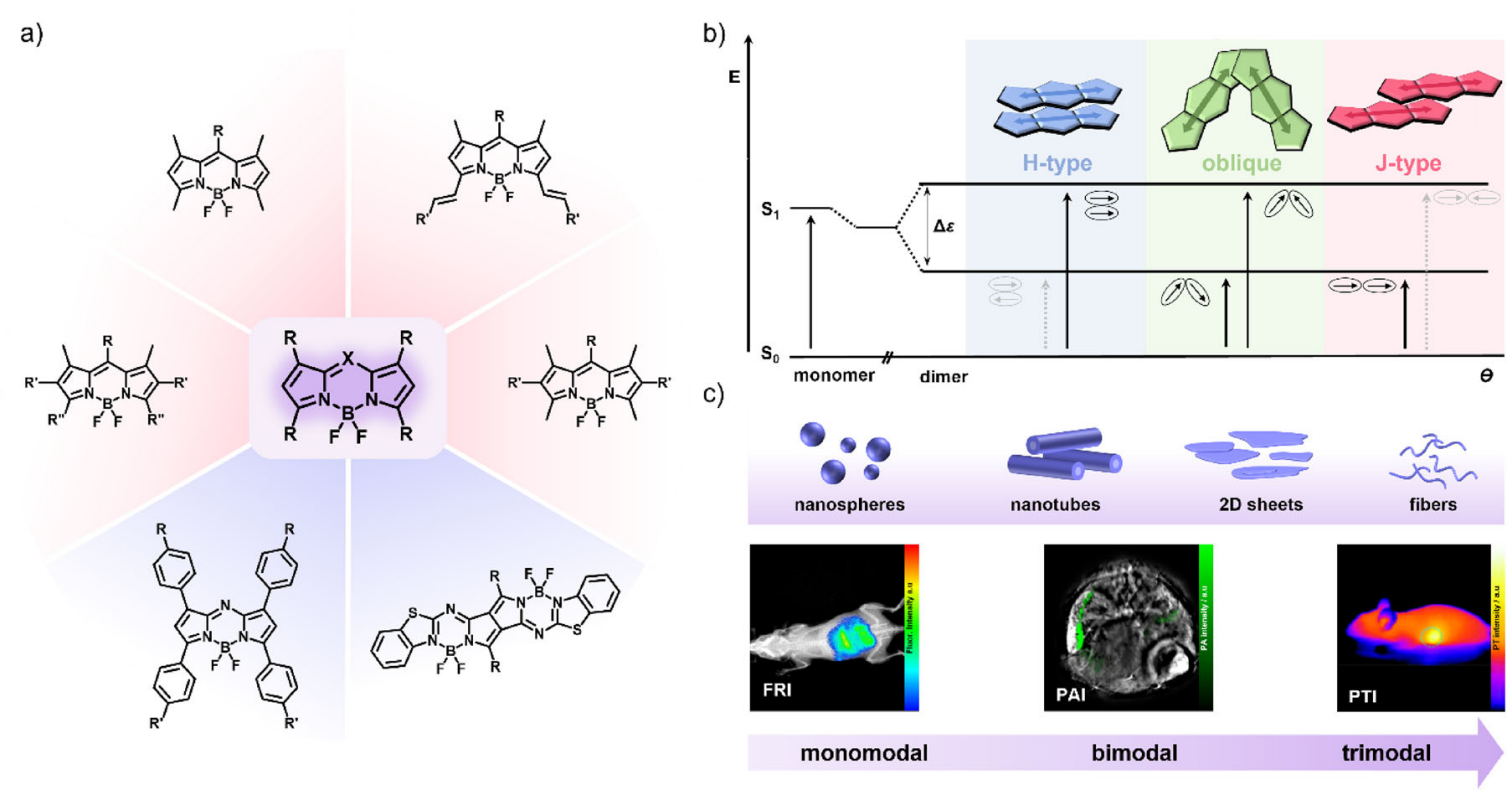
a) Representative chemical structures of (aza)BODIPYs that undergo self‐assembly. b) Schematic representation of Kasha's exciton coupling theory and c) of the different possible nanostructures or (multimodal) imaging techniques, respectively. Adapted from Ref. 39 Copyright 2025 Wiley‐VCH and Ref. 79 Copyright with permission from the Royal Society of Chemistry.

In this minireview, we classify (aza)BODIPY dyes according to their molecular stacking modes and aggregate types in aqueous media, correlating these with their multimodal imaging capability (Scheme 1). Examples involving carriers to encapsulate BODIPYs are beyond the scope of this review. We highlight the relevance of supramolecular self‐assembly for biomedical imaging and rationalize the relationship between molecular design, packing modes in the assembled state—which determines exciton coupling (H‐, J‐, and oblique‐type) and photophysical behavior—and imaging modality. Furthermore, we discuss the design criteria for (aza)BODIPY‐based CAs to advance from monomodal to trimodal imaging, aiming to realize comprehensive multimodal applications. To the best of our knowledge, no review has yet systematically explored the connection between molecular design, photophysical properties, and self‐assembly characteristics of (aza)BODIPY dyes in aqueous media, properties that decisively impact their imaging capabilities. Advancing CAs that support multiple imaging modalities is essential for achieving a deeper understanding of biological processes at the molecular level. By highlighting the underexplored potential of pathway complexity—where a single CA can give rise to multiple species with distinct photophysical properties—this minireview aims to inspire further research into versatile systems for multimodal imaging.

## 2 General Considerations About the Self‐Assembly Behavior of (aza)BODIPY Dyes

Owing to their planar and preorganized *π*‐system, (aza)BODIPYs are well known to self‐assemble in solution through stacking of the dipyrromethene core, often stabilized by secondary interactions, as documented in recent reviews.^[^ ^1^, ^2^ ^]^ In analogy to other functional organic dyes, (aza)BODIPYs can exhibit three main types of excitonic coupling according to Kasha's exciton theory: H‐type, J‐type, or oblique‐type (Scheme 1b).^[^ ^38^ ^]^ In all cases, excitonic coupling leads to a splitting of the excited state into a higher‐ and a lower‐energy exciton level; however, the relative orientation of the transition dipole moments determines which of these transitions is optically allowed. H‐type aggregates, characterized by parallel alignment of the transition dipole moments (face‐to‐face), show blue‐shifted absorption bands and typically quenched fluorescence. In this arrangement, the transition to the lower‐energy exciton state is optically forbidden due to destructive cancellation of the transition dipole moments, while only the higher‐energy transition is allowed. In contrast, J‐type aggregates, arising from a slipped molecular arrangement (head‐to‐tail), display red‐shifted absorption and often enhanced emission, a feature particularly attractive for NIR bioimaging. Here, the transition to the higher‐energy exciton state is optically forbidden due to dipole cancellation, whereas the lower‐energy transition is allowed. Oblique‐type aggregates are less common and display both red‐ and blue‐shifted absorption maxima compared to the monomer.^[^ ^39^ ^]^ In this coupling motif, the transition dipole moments adopt a non‐parallel, oblique orientation, such that neither purely constructive nor purely destructive interference occurs and consequently, in contrast to H‐ and J‐type aggregates, both excitonic transitions remain partially allowed. Kasha described this arrangement as a third limiting excitonic coupling case, ideally occurring for a near‐perpendicular dipole orientation.^[^ ^38^ ^]^ However, theoretical studies show that this ideal behavior is only observed within a very narrow range of oblique angles, making the spectral response extremely sensitive to small geometric deviations and thereby explaining the rare occurrence of this packing motif.^[^ ^40^, ^41^ ^]^ Such distinct types of exciton coupling are frequently observed in *π*‐conjugated systems, where subtle changes in molecular packing strongly influence the resulting photophysical properties. Importantly, self‐assembly is not necessarily confined to a single packing motif; the same monomer can give rise to multiple aggregated species under different conditions, a phenomenon known as pathway complexity. This refers to the ability of a single molecule to self‐assemble into more than one final structure, reflecting the interplay between kinetically and thermodynamically controlled species.^[^ ^42^, ^43^ ^]^ Kinetic supramolecular assemblies typically form rapidly under given conditions and represent metastable states trapped in local energy minima. Over time—if sufficient energy is provided—these kinetic species often transform into more stable thermodynamic aggregates, which correspond to the global energy minimum and typically dominate after equilibration. Based on these two limiting assembly regimes, pathway complexity can arise through either competitive or consecutive pathways: in the former, kinetic and thermodynamic aggregates compete for the same monomeric species, whereas in the latter, the kinetic state serves as a precursor to the thermodynamic state, for example through structural rearrangements or the formation of higher‐order architectures. The dominance of either pathway is highly sensitive to external parameters such as solvent, concentration, temperature, and mechanical perturbation.^[^ ^42^ ^]^ It should be noted that, in biological applications, pathway complexity must be carefully considered to avoid misinterpreting imaging signals that may arise from distinct, coexisting aggregate states.

**Figure 1:**
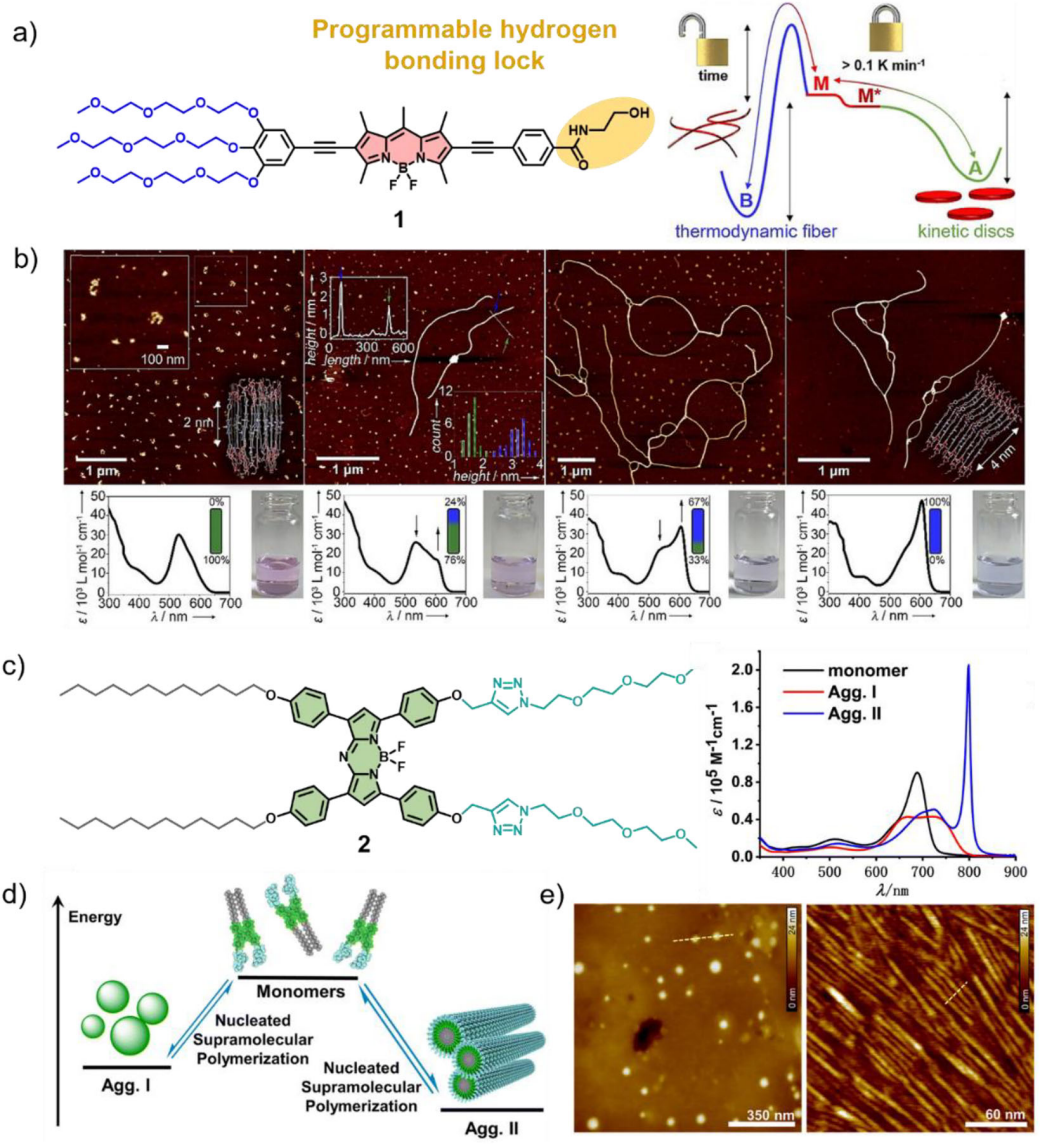
a) Chemical structure of **1** and the corresponding energy landscape illustrating its aggregation pathways. b) Time‐dependent AFM studies with corresponding UV/Vis spectra. Reprinted with permission from Ref. 44 Copyright 2021 Wiley‐VCH. c) Chemical structure of **2** and corresponding UV/Vis absorption spectra in the monomeric and different aggregated states. d) Schematic representation of the competitive pathway of Agg. I and Agg. II. e) AFM images of Agg. I (left) and Agg. II (right). Reprinted with permission from Ref. 45 Copyright 2017 Wiley‐VCH.

In recent years, detailed mechanistic studies on different (aza)BODIPY dyes have revealed the propensity of these molecules to exhibit pathway complexity. In a relevant example, our group reported an amphiphilic BODIPY dye **1** featuring a hydrogen‐bonding lock introduced via an N‐(2‐hydroxyethyl) amide head group (Figure 1a).^[^ ^44^ ^]^ This moiety forms a pseudocycle through intramolecular hydrogen bonding, giving rise to a “locked” monomer species (M*). In aqueous solution, M* initially assembles into kinetically controlled discoidal H‐type aggregates (A), which gradually transform into thermodynamically favored fibrillar J‐type aggregates (B) through intermolecular hydrogen bonding (Figure 1b). Kinetic analysis showed that A and B represent competitive pathways, while further mechanistic studies revealed isodesmic polymerization for A (Δ*G* = −44.4 kJ mol^−^^1^) and cooperative polymerization for B (Δ*G* = −49.1 kJ mol^−^^1^). Importantly, the length of the 1D fibers of B could be precisely tuned from ∼110 nm to ∼1.2 µm using a seeded living supramolecular polymerization (LSP) approach.

The competition between kinetic and thermodynamic self‐assembly pathways has also been observed for aza‐BODIPY dyes. In a representative example, Chen, Würthner, and co‐workers designed an amphiphilic aza‐BODIPY **2** that exhibits two competing pathways in methanol.^[^ ^45^ ^]^ Upon cooling, **2** initially forms metastable nanoparticles (Agg. I) with broadened absorption, which subsequently convert into J‐type aggregates (Agg. II) showing narrow and intense absorption bands with a pronounced bathochromic shift of around 100 nm, indicating molecular rearrangement toward higher order with strong excitonic coupling (Figure 1c–e).

These examples highlight that the combination of intrinsic structural tunability, assembly‐driven photophysical modulation, and compatibility across multiple modalities renders (aza)BODIPY derivatives highly promising as adaptable and efficient CAs for biomedical imaging. Additionally, the coexistence of multiple assemblies derived from the same monomer provides further structural versatility and expands the functional diversity of supramolecular systems, for example enabling turn‐on performance.^[^ ^1^, ^2^, ^45^, ^46^ ^]^

## 3 (Aza)BODIPY‐Based Self‐Assemblies for Biomedical in Vivo Imaging

Based on the different packing arrangements and the resulting photophysical properties found for (aza)BODIPYs, this has led to a range of different functions, notably enhancing their potential for biomedical imaging applications.^[^ ^8^ ^]^ In the following sections, we have classified (aza)BODIPY derivatives depending on their type of packing within the supramolecular self‐assemblies and the resulting variety of imaging modalities.

### 3.1 H‐Type Aggregates

#### 3.1.1 Fluorescence Imaging in ACQ Systems

Fluorescence imaging (FI) is a non‐invasive optical imaging technique using planar illumination of the target at certain excitation wavelengths resulting in fluorescence emission. It is valued for its high sensitivity, multi‐labeling capability, stability, safety, and low cost, making it widely used to visualize physiological and pathological processes in living systems.^[^ ^47^ ^]^ However, in H‐type aggregates, face‐to‐face stacking of the chromophores leads to ACQ of emission.^[^ ^38^, ^48^ ^]^ Although this property appears detrimental for bioimaging applications, strategies to induce disassembly can trigger the recovery of the monomeric luminescence, which can be exploited to monitor biological and biomedical processes.^[^ ^49^ ^]^

**Figure 2:**
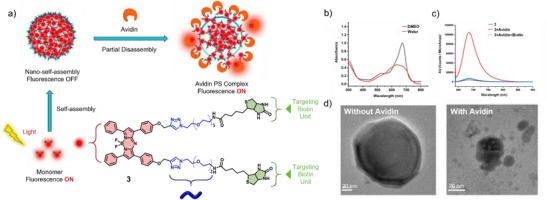
a) Schematic illustration of the switchable fluorescence activity that results from self‐assembly and partial disassembly processes of **3** triggered by avidin. b) UV/Vis spectra of **3** in DMSO and water. c) Emission spectra and d) HR‐TEM images showing the turn‐on fluorescence activity of **3** in the presence of avidin. Reprinted from Ref. 50 with permission from the Royal Society of Chemistry.

In this regard, Gogoi, and co‐workers synthesized an aza‐BODIPY biotin‐based derivative that forms non‐emissive spherical H‐type aggregates in aqueous media (Figure 2).^[^ ^50^ ^]^ In the presence of avidin—which has the ability to bind biotin molecules—the assemblies undergo partial disassembly and thus, enable NIR turn‐on fluorescence activity as demonstrated by FI (Figure 2a,b). However, the recovered fluorescence of avidin/**3** was markedly lower than that of the molecularly dissolved species (Figure 2c). The authors hypothesize that some monomers of **3** were released from the spherical assemblies due to strong avidin–biotin binding, while inner biotin units remained trapped by intra‐ and intermolecular linkages. This stabilization prevented complete nanosphere disruption, as corroborated by high‐resolution transmission electron microscopy (HR‐TEM) images (Figure 2d). Since tumor tissues are associated with the overexpression of various receptor proteins such as biotin receptors, these results signify the potential of the self‐assembly probes for cancer bioimaging applications.

**Figure 3:**
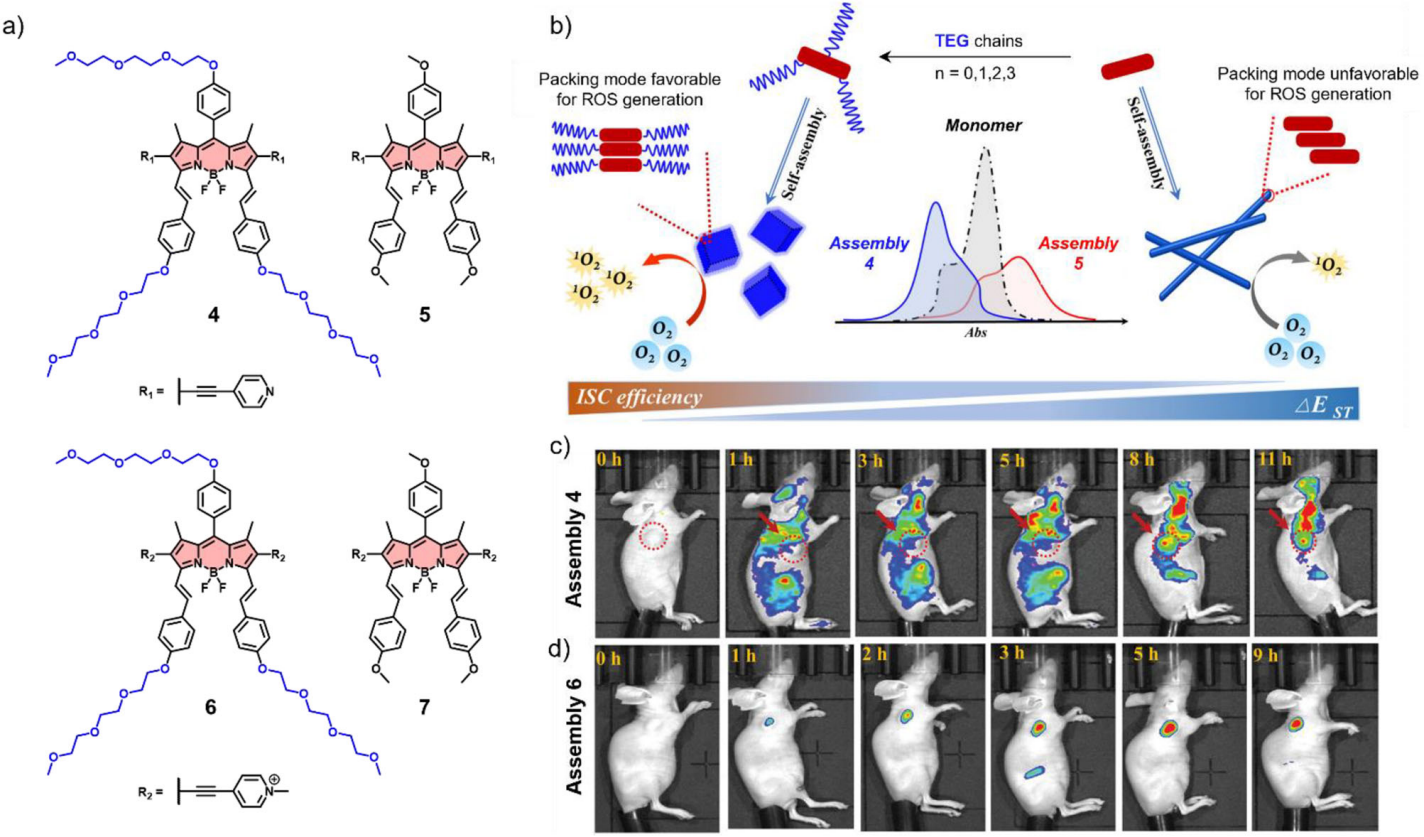
a) Chemical structures of **4–7**. b) Schematic illustration of the different packing modes of assemblies formed by **4** and **5** and their impact on the photophysical properties. In vivo FI of c) **4** and d) **6** in HeLa (cervical cancer cells) tumor‐bearing mice after tail vein injection. Reprinted from Ref. 52 Copyright 2023 American Chemical Society and Ref. 53 Copyright 2023 Wiley‐VCH.

In some cases, it has been observed that H‐type aggregation can induce substantial ACQ without fully quenching emission, leaving enough brightness for imaging applications.^[^ ^51^ ^]^ In this regard, Niu, Cui, Yang, and co‐workers developed heavy‐atom‐free BODIPY derivatives **4** and **5** by introducing varying numbers of triethylene glycol (TEG) chains as bulky hydrophilic substituents (Figure 3).^[^ ^52^ ^]^ These modifications influenced intermolecular interactions, packing modes and self‐assembly behaviors, resulting in H‐type aggregates for **4** driven by interlocking side‐chain interactions, while **5** assembled into J‐type aggregates dominated by *π*–*π* interactions (Figure 3a,b). Furthermore, assemblies of **4** achieved a 75‐fold higher ^1^O_2_ generation rate than **5** due to enhanced intersystem crossing (ISC). In vivo, **4** demonstrated strong tumor‐site fluorescence within 6 h (Figure 3c), underscoring its excellent tumor‐targeting ability and potential for FI‐guided PDT. In another example, the same group further introduced a charged moiety by designing pyridinium derivatives **6** and **7** as ultra‐small nano‐photosensitizers with tumor‐specific accumulation and renal clearance.^[^ ^53^ ^]^ Due to the dual cationic features and a hydrophilic TEG coating, **6** exhibited enhanced stability, prolonged circulation, efficient tumor targeting, and cellular uptake. Additionally, a fluorescence signal appeared at tumor sites as early as 30 min post‐injection and intensified over time, confirming the efficient tumor accumulation of **6** (5.6 nm). In contrast, **4** (90 nm) also accumulated in the tumor stroma via the EPR effect, but with markedly slower kinetics (up to 8 h) and a lower signal‐to‐background (Figure 3d). Collectively, these findings underscore the superior generation efficiency of ROS and tumor‐targeting performance of **6**.

#### 3.1.2 Fluorescence Imaging in AIE Systems

**Figure 4:**
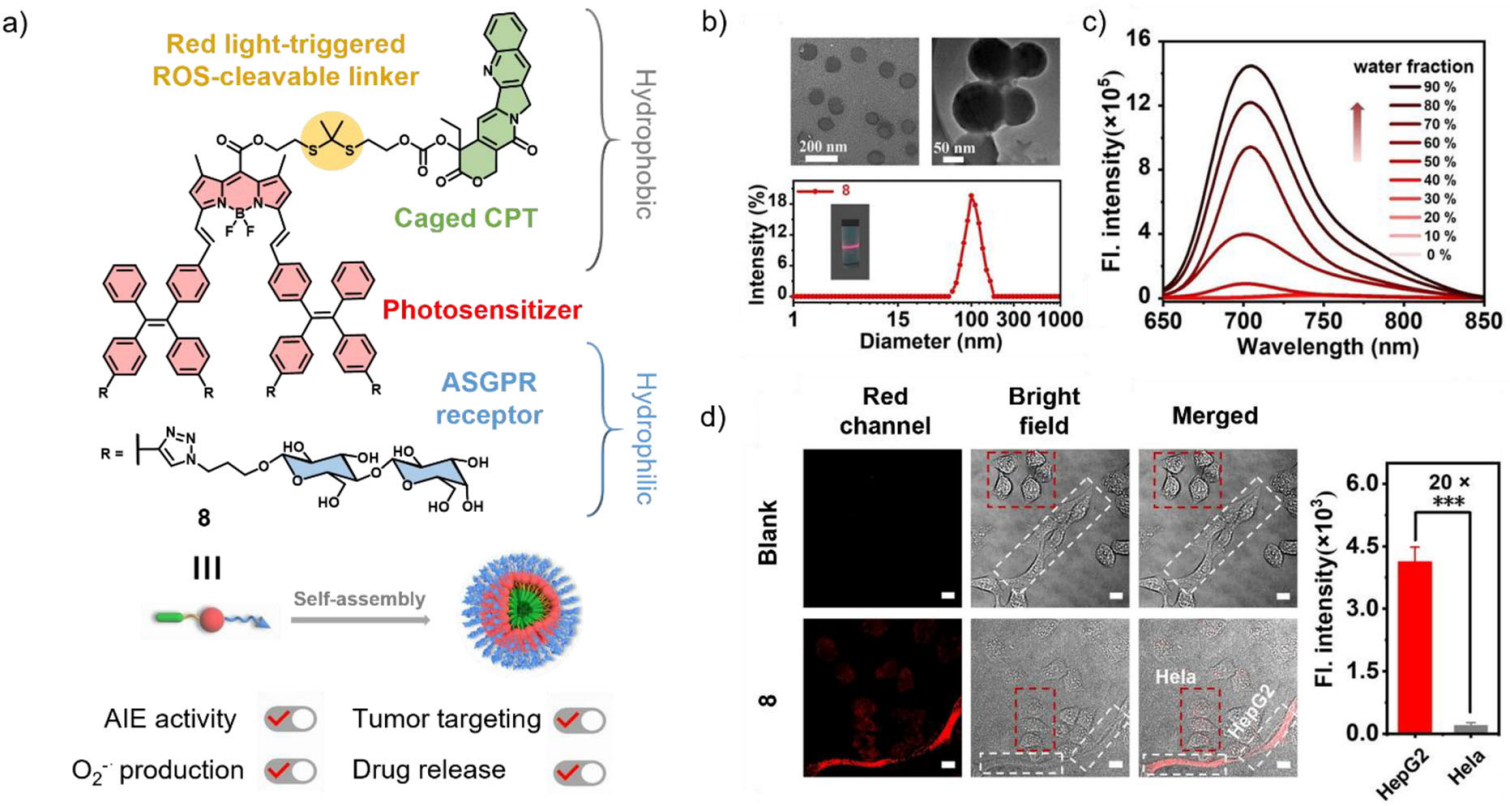
a) Chemical structure of **8** assigning the different molecular functionalities. b) TEM images and DLS results of the aqueous assemblies of **8**. c) Emission spectra with different ratios of DMSO/water. d) Confocal fluorescence images of **8** in an in vitro coculture model with HepG2 and HeLa cells. Reprinted from Ref. 56 Copyright 2025 Wiley‐VCH.

As previously noted, H‐type aggregates are generally associated with ACQ; therefore, controlled disassembly of the aggregates offers a means to restore emission. Alternatively, fluorescence enhancement can be achieved through the incorporation of aggregation‐induced emission (AIE) substituents, which effectively eliminate the ACQ that is typically observed in (aza)BODIPY derivatives.^[^ ^54^ ^]^ Among the various AIE‐active fluorophores, tetraphenylethylene (TPE) is particularly well established for its strong luminescent properties and is frequently employed to improve the emissive performance of such systems.^[^ ^55^ ^]^ In this context, Xing and co‐workers combined a lactose‐substituted BODIPY‐TPE derivative **8** with camptothecin (CPT) and an NIR light‐triggered ROS‐cleavable linker (Figure 4a).^[^ ^56^ ^]^ In aqueous solution, **8** exhibits a blue‐shift in its absorption band relative to the monomer species, indicative of H‐type aggregate formation. While almost no fluorescence is observed in pure DMSO, the addition of water distinctly enhanced the fluorescence intensity (*Φ*_FL_ = 0.5% to 29.3%), demonstrating a typical AIE characteristic (Figure 4b,c). This enhancement arises from the rigidified molecular structure of **8** in its aggregated state, thereby suppressing non‐radiative decay pathways. Furthermore, the twisted molecular conformation induced by hydrogen bonding and van der Waals interactions reduces *π*–*π* stacking through non‐parallel *π*‐orbital alignment and steric repulsion between neighboring bulky CPT groups, further promoting strong fluorescence emission. Considering the conjugation of lactose moieties to (aza)BODIPYs, this not only imparts excellent water solubility but also enables efficient asialoglycoprotein receptor (ASGPR‐)mediated targeting and uptake in HepG2 liver cancer cells, resulting in outstanding imaging capability for precision tumor therapy (Figure 4d).^[^ ^57^ ^]^ In addition, the regulation of carbohydrate–carbohydrate interactions between lactose units in the aggregated state contributes to the superior ROS generation of **8**. Overall, these properties endow **8** with powerful bioimaging performance as well as therapeutic potential, providing an effective platform for real‐time tracking, precise PDT, and photoactivated drug release in murine cancer treatment.

### 3.2 J‐Type Aggregates for Monomodal Imaging

Compared to H‐type aggregates, J‐type assemblies are particularly attractive because their slip‐stacked arrangement induces red‐shifted absorption and emission, often facilitating the extension into the NIR region. This can be achieved by varying substitution patterns at the (aza)BODIPY core, effectively modulating self‐assembly, as intermolecular interactions and chromophore packing determine the photophysical behavior. To this end, various molecular design strategies have recently been explored to promote slipped stacking resulting in J‐type aggregation, e.g., through the attachment of bulky substituents at the (aza)BODIPY core.^[^ ^58^, ^59^, ^60^, ^61^ ^]^

#### 3.2.1 Fluorescence Imaging in ACQ Systems

**Figure 5:**
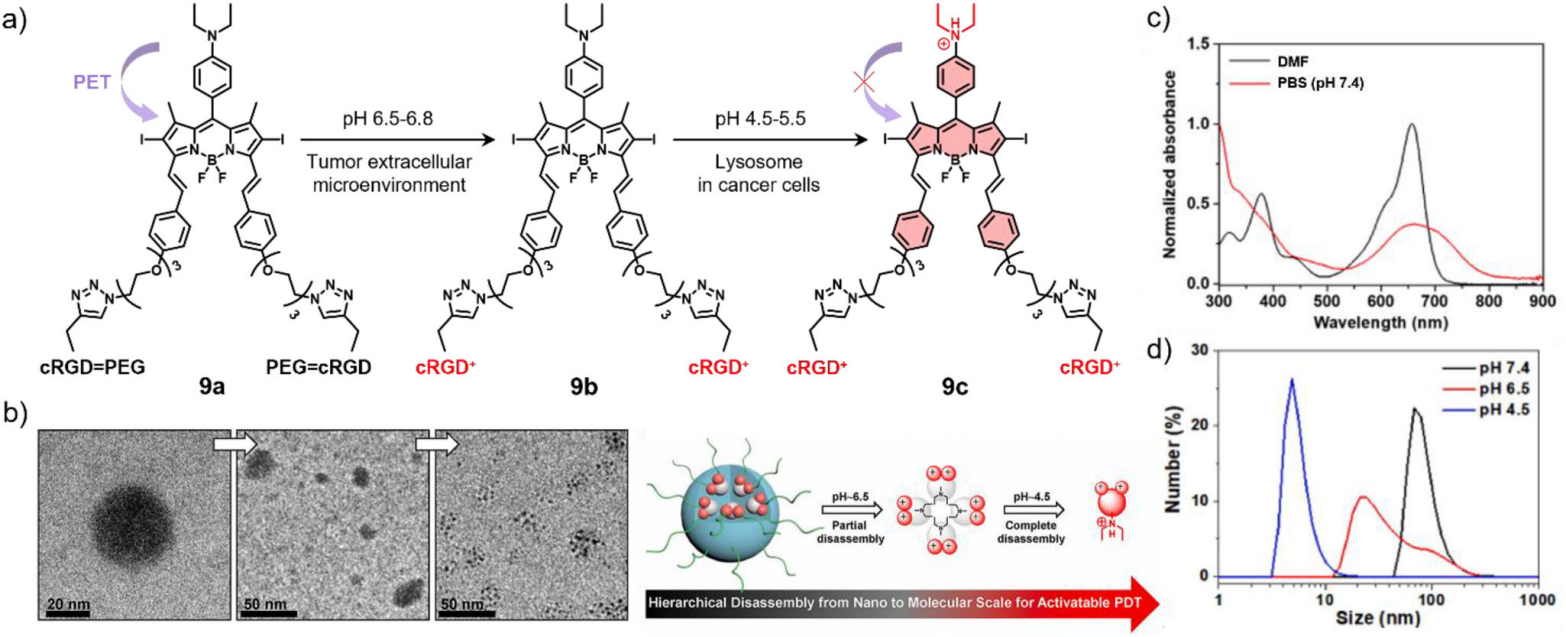
a) Chemical structure of **9** and its response to pH changes. b) Schematic illustration of the acid‐triggered hierarchical disassembly of **9** demonstrated by TEM images. c) Absorption spectra of **9a** in DMF and PBS buffer. d) DLS results at different pH values. Reprinted from Ref. 62 with permission from Elsevier.

Although J‐type aggregates are generally expected to exhibit enhanced emission properties, ACQ is still frequently observed in aqueous media. In order to recover the luminescence characteristics, Zhang, Zhao, and co‐workers developed an RGD‐functionalized BODIPY derivative **9** that undergoes acid‐triggered hierarchical disassembly of J‐type aggregates for enhanced tumor penetration and activatable PDT (Figure 5a,b).^[^ ^62^ ^]^ To improve solubility and prolong circulation, cleavable polyethylene glycol (PEG) entities were introduced, enabling tumor accumulation via the EPR effect.^[^ ^63^ ^]^ In the mildly acidic TME (pH ∼6.5), hydrolysis of the Schiff base bond between PEG and cRGD (**9a**) initiates a first‐stage size reduction, where PEG detachment exposes protonated cRGD peptides (**9b**). This leads to Coulombic repulsion within the hydrophobic core, partial nanoparticle disassembly, enhanced tumor penetration, and selective recognition of integrin *α*_v_ *β*_3_–overexpressing cancer cells. Following endocytosis, the lysosomal acidity (pH ∼4.5) induces a second‐stage size reduction, yielding complete disassembly into monomers **9c**. The hierarchical disassembly process was corroborated by dynamic light scattering (DLS) and TEM analysis (Figure 5c,d). Protonation of the diethylamino group suppresses photoinduced electron transfer (PET), thereby restoring fluorescence for tumor imaging and enabling efficient ROS generation at the molecular level, ultimately enhancing PDT efficacy while minimizing off‐target phototoxicity. In addition to its mildly acidic pH, another characteristic of the TME is the overexpression of specific enzymes. Consequently, the incorporation of enzyme‐cleavable linkers in the molecular design can enable enzyme‐triggered disassembly and thus, provides an alternative strategy for achieving controlled FI.^[^ ^64^ ^]^

#### 3.2.2 Photothermal Imaging

Although J‐type aggregates are often emissive, in aqueous environments and especially in the NIR region, non‐radiative relaxation dominates due to: 1) the energy‐gap law which states that the rate of non‐radiative decay increases as the energy gap between the excited and ground states decreases; 2) strong vibrational coupling with the solvent; and 3) excitonic delocalization that channels energy into photons.^[^ ^65^ ^]^ As a result, these systems efficiently convert absorbed light into heat rather than emitting brightly, enabling photothermal imaging (PTI). This non‐invasive technique visualizes local temperature changes using infrared (IR) cameras or other thermal detectors, allowing real‐time and highly sensitive mapping of heat generation. PTI is therefore particularly useful for assessing photothermal agents, monitoring therapeutic outcomes, and studying heat‐induced biological responses.^[^ ^66^ ^]^ In this context, platinum (II) complexes are particularly attractive owing to their strong spin–orbit coupling, which promotes ultrafast ISC from singlet to triplet states. Once in the triplet state, the square‐planar geometry provides access to metal‐centered d–d states, which are weakly emissive and thus, facilitate non‐radiative decays. These combined effects suppress fluorescence while enhancing thermal energy release, making Pt‐containing systems highly suitable for bioimaging as well as photoinduced therapeutic applications.^[^ ^67^ ^]^

**Figure 6:**
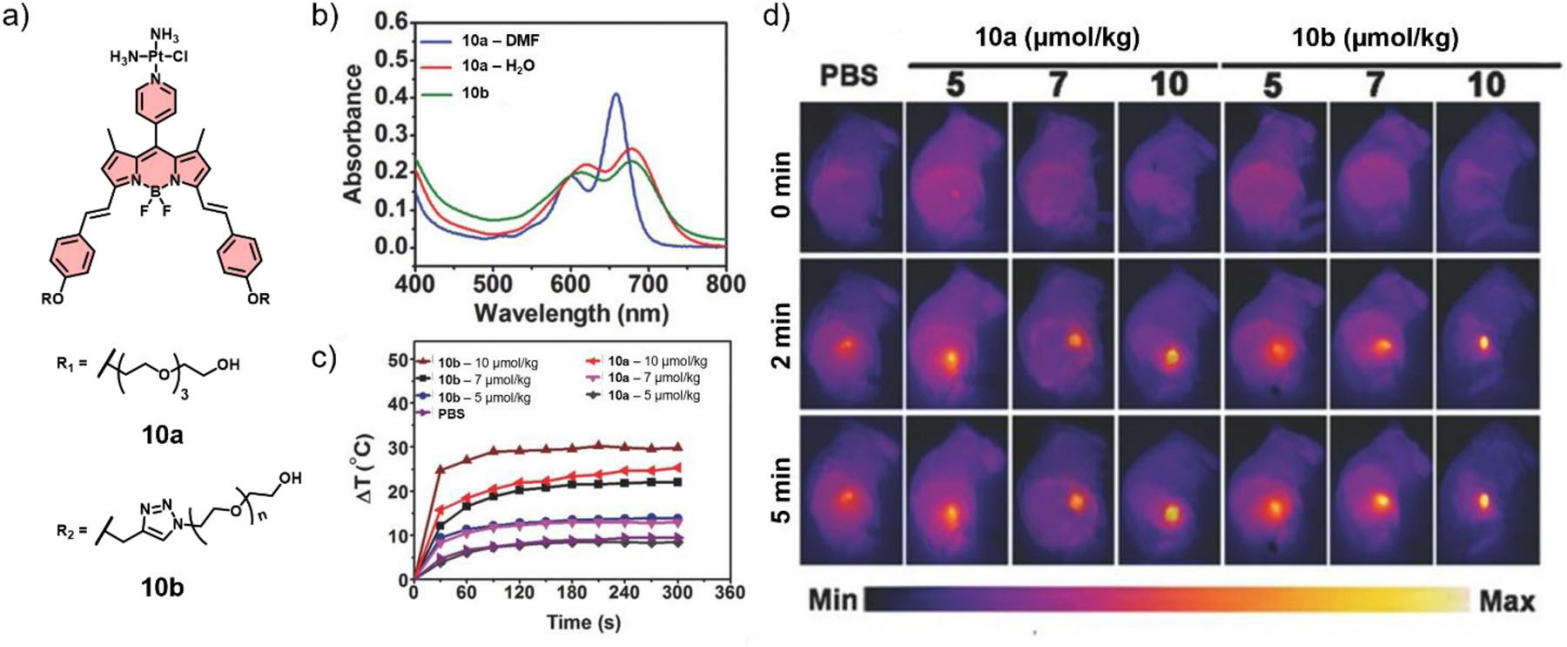
a) Chemical structures of **10a** and **10b**. b) Absorption spectra in different solvents. c) Time‐dependent temperature elevation and d) infrared thermography at the tumor site of the mice injected with various doses of **10a** or **10b**, respectively, under 5 min irradiation (660 nm, 0.5 W cm^−2^). Reprinted from Ref. 68 Copyright 2016 Wiley‐VCH.

Chen and co‐workers presented two platinum‐modified BODIPYs, without (**10a**) and with PEGylation (MW 5000) (**10b**), to assess a promising bifunctional platform for photoinduced tumor ablation due to their combined photothermal and photodynamic properties (Figure 6a).^[^ ^68^ ^]^ Both compounds exhibited red‐shifted absorbance, suggesting the formation of J‐type aggregates (Figure 6b). However, PEGylation further improves the stability and resistance to photobleaching of these nanoparticles, making them more effective for in vivo tumor treatment. In contrast to **10a**, **10b** showed enhanced photothermal conversion efficiency (PCE) resulting from improved non‐radiative decays in its self‐assembled state and optimized hyperthermic effects, demonstrated by dose‐dependent tumor temperature elevation under IR irradiation in vivo (660 nm, 0.5 W cm^−2^) (Figure 6c,d). Overall, **10b** efficiently generates singlet oxygen and provides effective photothermal conversion, making it a promising approach for photoinduced tumor ablation.

**Figure 7:**
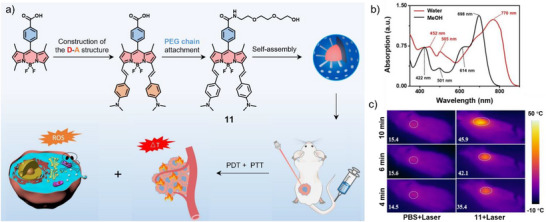
a) Schematic representation of the construction of D–A–D structure **11** and its PDT/PTT capability. b) Absorption spectra of **11** in methanol and water. c) Photothermal images of mice irradiated by 808 nm laser (1 W cm^−2^). Reprinted from Ref. 69 with permission from Elsevier.

Another strategy to reduce the energy gap between the electronic states and thus, promote non‐radiative decays, is the utilization of donor–acceptor–donor (D–A–D) architectures. Such systems have been extensively explored for PDT and PTT due to their advantageous electronic properties, shifting the absorption and emission bands into the NIR region.^[^ ^16^ ^]^ Following this design principle, Zhao, Jiang, Li, Wang, and co‐workers developed a PEGylated BODIPY **11** (Figure 7a).^[^ ^69^ ^]^ Upon addition of water, **11** undergoes J‐type aggregation with red‐shifted absorption (Figure 7b) and reduced emission attributed to *π*–*π* stacking and electron delocalization. However, upon NIR irradiation, **11** not only generates singlet oxygen but also dissipates energy as heat, thus providing a foundation for synergistic PDT and PTT (Figure 7c).

### 3.3 J‐Type Aggregates for Bimodal Imaging

While each molecular imaging technique offers distinct advantages, inherent limitations often compromise their accuracy and reliability in depicting specific biological processes. Consequently, most clinically applied CAs are restricted to a single modality with fixed properties, hindering cross‐comparison between techniques. Moreover, key physicochemical parameters―such as size, shape, surface charge, and morphology―critically influence cellular uptake, tissue extravasation, biodistribution, bioavailability, and clearance, further limiting their applicability. Because these processes span multiple scales, from single cells to whole organisms, and evolve both spatially and temporally, the advancement of multimodal and multiscale imaging strategies is vital for unraveling the complex biological interactions underlying health and disease.^[^ ^8^, ^70^ ^]^ However, designing materials suitable for multimodal imaging poses additional challenges, as each modality demands distinct photophysical requirements; for instance, a high fluorescence quantum yield is advantageous for FI but detrimental for PAI. Therefore, creating a single organic material capable of functioning efficiently across multiple biomedical imaging modalities requires a careful balance of optical and physicochemical properties. Leveraging the complementary strengths of different modalities enables more accurate and comprehensive visualization of biological processes at the molecular level. In this regard, the discovery of J‐type aggregates has opened new possibilities for achieving multimodal imaging capabilities.

#### 3.3.1 FI Combined with PAI

Photoacoustic imaging (PAI) is an emerging real‐time non‐invasive modality that exploits the photoacoustic effect for dye‐specific molecular imaging. It combines pulsed laser excitation with ultrasonic detection, where short multispectral laser pulses are absorbed by the target and converted into heat through non‐radiative decay, inducing thermoelastic expansion through vibrational or collisional relaxation. The resulting acoustic waves are detected and reconstructed into an absorption distribution map. Unlike conventional FI, which is limited to micrometer‐scale depths, PAI achieves tissue penetration of several centimeters with high spatial resolution, as sound scatters much less than light.^[^ ^71^, ^72^ ^]^ Over the past years, several CAs have evolved for PAI in order to increase resolution and specificity, including metal nanoparticles,^[^ ^73^ ^]^ quantum dots,^[^ ^74^ ^]^ and small organic molecules.^[^ ^75^ ^]^ In recent years, the combination of FI and PAI has emerged as a promising multimodal strategy, enabling the simultaneous acquisition of synergistic information for comprehensive biomedical visualization. FI offers high sensitivity and excellent spatial resolution for tracking biological processes at the cellular and molecular levels, while PAI provides deeper tissue penetration and high contrast based on optical absorption. However, the combination of these methods is challenging due to opposed photophysical requirements. Thus, designing an organic material suitable for multimodal biomedical imaging requires finely tuning its photophysical properties to satisfy the distinct requirements of each technique.^[^ ^22^ ^]^

**Figure 8:**
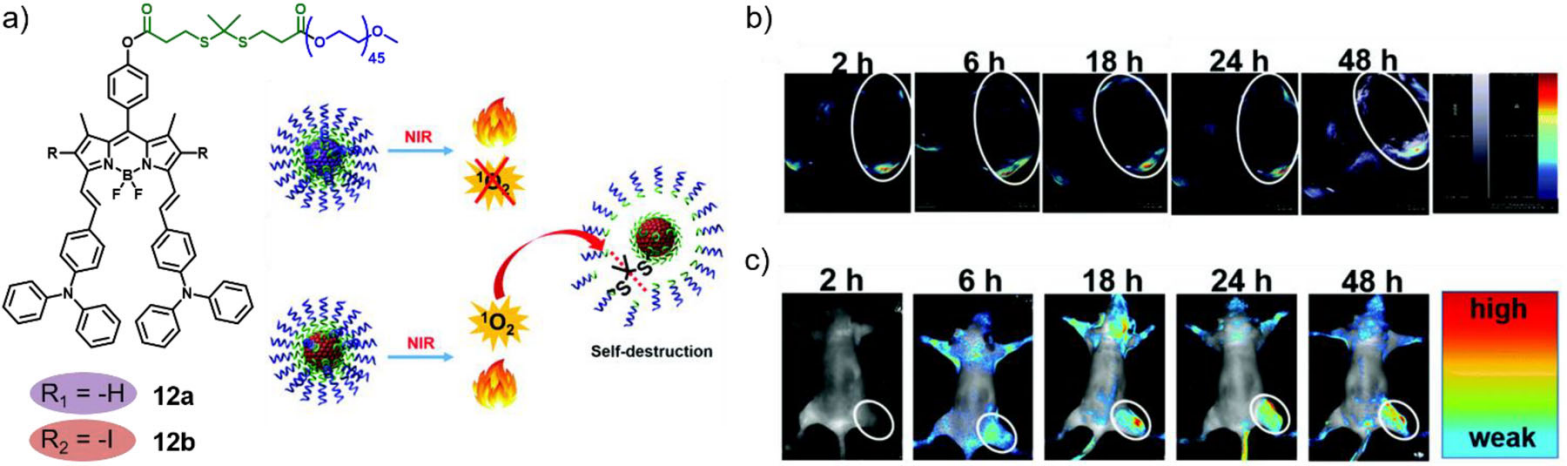
a) Schematic illustration of the self‐assembly and self‐destruction of **12a** and **12b**. b) PAI and c) FI of tumor tissues in vivo after tail vein injection at different timepoints. Reprinted from Ref. 76 Copyright 2019 American Chemical Society.

In this regard, Zhang, Xie, and co‐workers functionalized a styryl‐substituted BODIPY **12** with PEG and a ROS‐responsive thioketal linker (Figure 8a).^[^ ^76^ ^]^ The resulting nanoparticles of **12a** and **12b** served as dual‐mode imaging agents for NIR fluorescence and photoacoustic modalities. Under light irradiation, the nanoparticles generated both heat and ROS, which further cleaved the thioketal bonds, inducing nanoparticle disassembly and facilitating controlled drug release. The incorporation of iodine (**12b**) enhanced ISC and ROS generation but reduced fluorescence due to heavy‐atom effects and ACQ.^[^ ^77^ ^]^ In vivo, **12b** assemblies demonstrated time‐dependent photoacoustic signals, with peak tumor accumulation at 6 h post‐injection (Figure 8b). FI revealed delayed fluorescence appearance at tumor sites, reflecting nanoparticle disassembly and alleviation of the quenching effect (Figure 8c). Collectively, **12b** achieved synergistic PTT/PDT under a single light source, with concurrent imaging capabilities and ROS‐triggered self‐destruction for controlled drug delivery.

**Figure 9:**
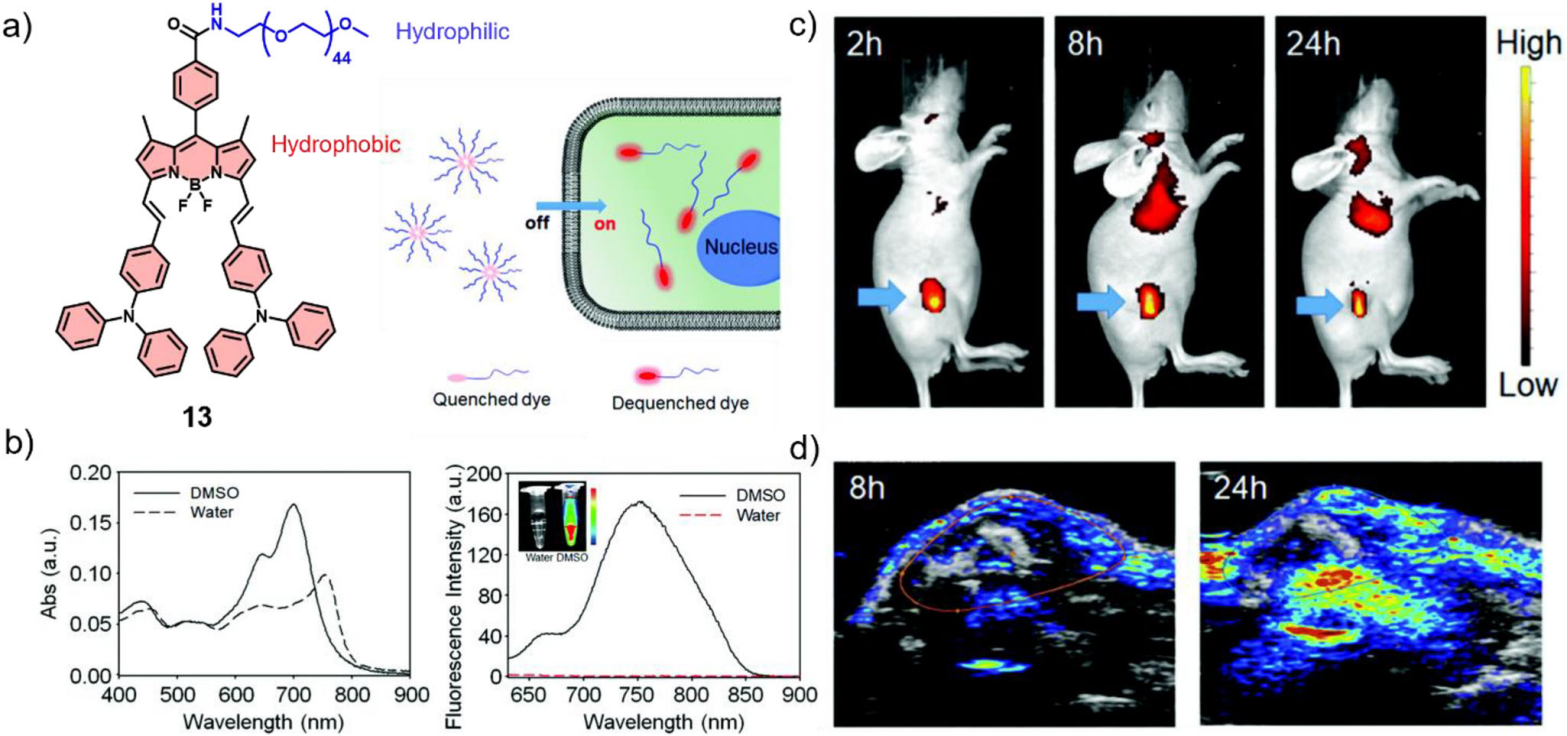
a) Chemical structure and fluorescence turn‐on properties of **13**. b) Absorption and emission spectra in DMSO and water. In vivo c) FI and d) PAI of tumor‐bearing mice. Reprinted from Ref. 22 with permission from the Royal Society of Chemistry.

In a complementary approach, a structurally related BODIPY derivative **13** was reported by Chen, Zhang, Ting, and co‐workers exhibiting ideal absorption and emission characteristics in the NIR region with a quantum yield of 10.1% in DMSO (Figure 9a,b).^[^ ^22^ ^]^ While fluorescence emission was strongly attenuated due to ACQ, once internalized by cells, disassembly of the nanoparticles released monomeric **13**, restoring the fluorescence signal and enabling enhanced imaging both in vitro and in vivo (Figure 9c). Furthermore, the strong NIR absorption of the assembled nanoparticles rendered them suitable for PAI, with signal intensities increasing over time and peaking at 24 h post‐injection (Figure 9d), consistent with efficient tumor targeting and accumulation.

#### 3.3.2 FI Combined with PTI

**Figure 10:**
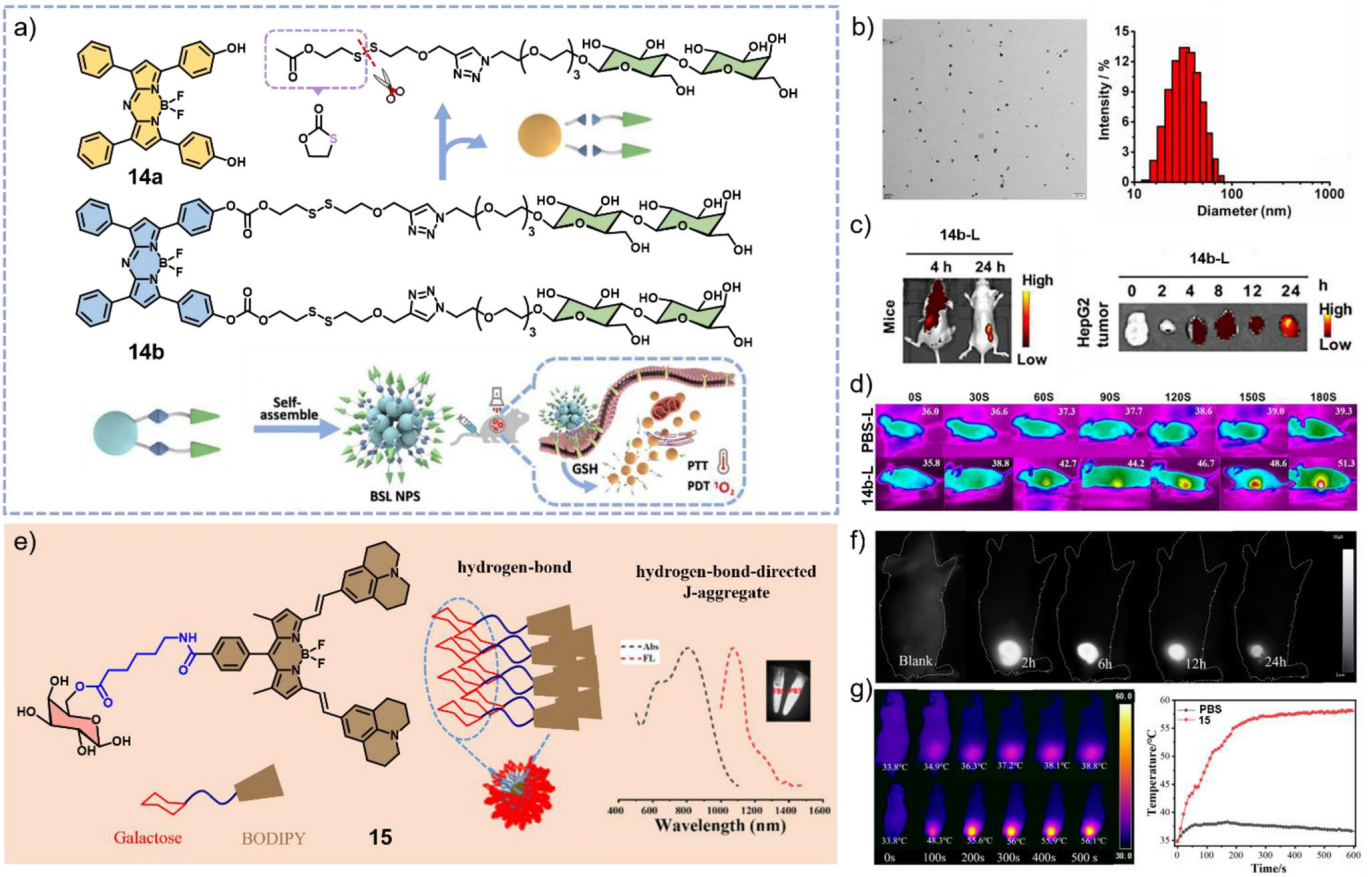
a) Chemical structures and schematic representation of the self‐assembly process and GSH‐responsiveness of **14a** and **14b**. b) TEM images (scale bar: 200 nm) and DLS results of **14b**. c) FI and d) Infrared thermography at the tumor sites. Reprinted from Ref. 78 with permission from Elsevier. e) Schematic representation of the self‐assembly process of **15**. f) FI and g) PTI with corresponding temperature curves of HepG2 (liver cancer cells) tumor‐bearing mice after injection of **15**. Reprinted from Ref. 24 with permission from Elsevier.

Recent work of Pei, Lui, Pei, and co‐workers has demonstrated carrier‐free nanoparticles consisting of amphiphilic glycosylated aza‐BODIPY **14b** (Figure 10a,b) with the ability to self‐assemble in aqueous media. This approach significantly enhanced cellular uptake by the selective targeting of HepG2 cells via galactose receptors on the cell membrane.^[^ ^78^ ^]^ Once reaching the TME, disulfide bonds in **14b** are cleaved by GSH, releasing **14a**. In vivo and ex vivo FI of HepG2 tumor‐bearing BALB/c nude mice after intravenous injection of **14b** displayed a red fluorescence signal that gradually intensified in the tumor region over time, indicating GSH‐mediated release of **14a** (Figure 10c). Furthermore, this species produces ROS and heat simultaneously under 685 nm NIR irradiation, thereby inducing synergistic photodynamic and photothermal cytotoxicity with a singlet oxygen quantum yield of *Φ*_Δ_ = 0.32 and a tumor‐site temperature increase of 15.5 °C, respectively (Figure 10d).

In another relevant example, Yan and co‐workers reported a saccharide‐functionalized aza‐BODIPY derivative **15** that forms J‐type aggregates through intermolecular hydrogen bonding and *π*–*π* stacking interactions in water (Figure 10e), enabling fluorescence emission in the second near‐infrared (NIR‐II) window.^[^ ^24^ ^]^ Besides NIR‐II FI (Figure 10f), the nanoparticles of **15** exhibited strong photothermal performance under 808 nm laser irradiation, reaching a high PCE of 55% (Figure 10g). These results highlight **15** as promising agent for galactose‐targeted NIR‐II imaging and efficient PTT, enabling precise tumor diagnosis and treatment.

### 3.4 J‐Type Aggregates for Trimodal Imaging

While bimodal imaging systems have demonstrated significant advantages by combining complementary modalities, extending this approach to trimodal imaging remains scarce. This strategy could offer a more comprehensive understanding of biological processes by further compensating for the inherent limitations of individual techniques.

**Figure 11:**
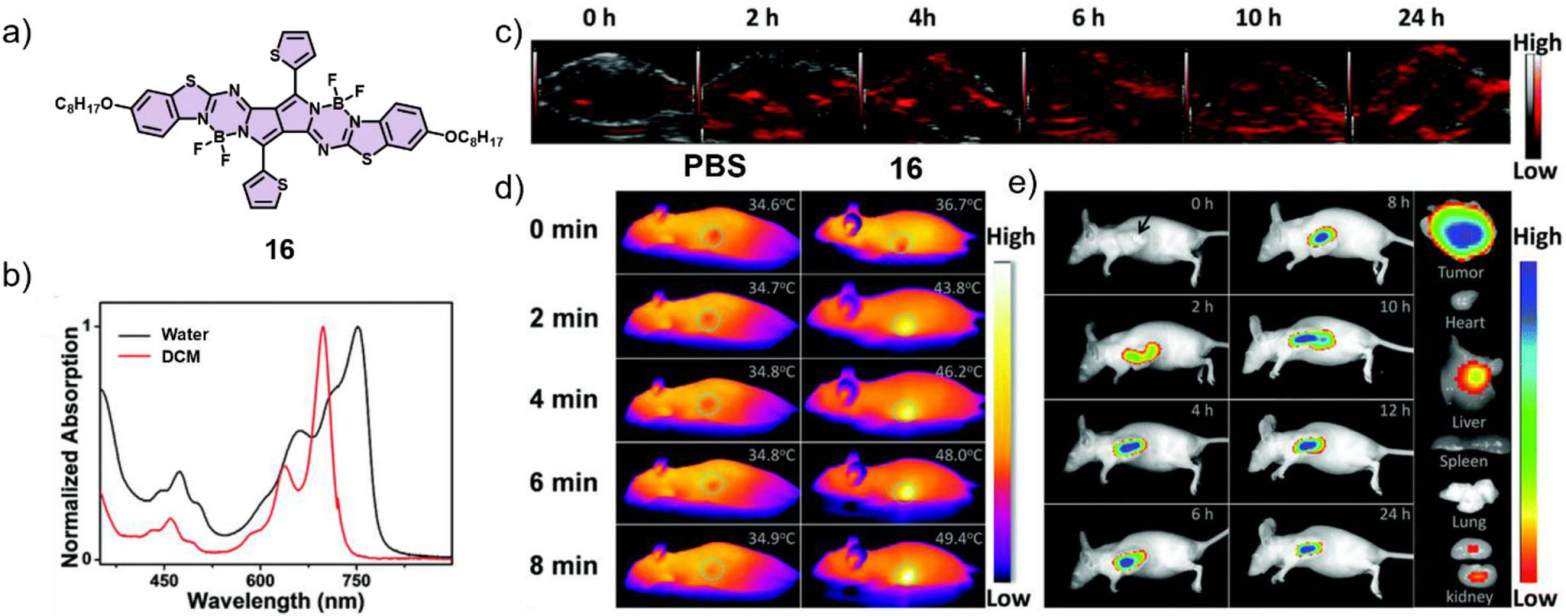
a) Chemical structure of **16** and b) absorption spectra in DCM and water. In vivo c) PAI at tumor sites after intravenous injection, d) PTI of tumor‐bearing mice exposed to laser irradiation for 8 min after injection of PBS or **16**, respectively, and e) FI of living mice bearing xenograft HeLa tumors. Reprinted from Ref. 79 with permission from the Royal Society of Chemistry.

As previously discussed, extending the *π*‐conjugated surface represents an effective strategy to red‐shift absorption into the NIR region. Following this approach, Sun, Shao, Dong, and co‐workers developed a diketopyrrolopyrrole (DPP)‐derived aza‐BODIPY derivative **16** that displays strong NIR absorption and emission in aqueous media (Figure 11a,b).^[^ ^79^ ^]^ Both in vitro and in vivo studies demonstrated the suitability for PAI (Figure 11c) as well as pronounced NIR fluorescence and exceptional photothermal performance, with rapid temperature elevation under 730 nm laser irradiation (Figure 11d,e). Compound **16** also proved successful for PTT, exhibiting robust tumor accumulation, efficient ablation capability, superior photothermal stability, and minimal dark cytotoxicity.

**Figure 12:**
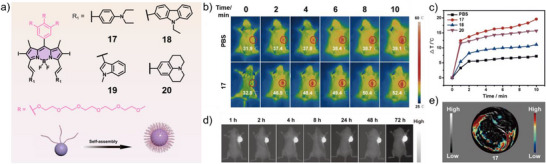
a) Chemical structures of **17–20** forming water‐stable nanoparticles. b) Infrared thermal images and c) corresponding temperature elevation curves. In vivo d) FI and e) PAI of **17** at the tumor sites recorded for 4T1 (breast cancer cells) tumor‐bearing mice under 808 nm laser irradiation (1.0 W cm^−2^). Reprinted from Ref. 80 with permission from the Royal Society of Chemistry.

According to the D–A–D design strategy, Li, Yin, and co‐workers synthesized a series of iodine‐substituted, hydrophilic BODIPY derivatives (**17**–**20**) bearing sterically demanding electron donors at the 3‐ and 5‐positions (Figure 12a).^[^ ^80^ ^]^ These structural modifications induced bathochromic shifts into the NIR region and promoted varying degrees of J‐type aggregation. Although the fluorescence quantum yields decreased markedly relative to the monomeric species, the nanoparticles retained NIR emission sufficient for imaging applications. Furthermore, the markedly different singlet oxygen quantum yields (9.33%–88.23%) suggest that free molecules remain within the nanoparticle cavities. The J‐type aggregation suppressed both radiative transitions and ISC, favoring non‐radiative decay pathways and resulting in substantial photothermal conversion. Except for compound **19**, all derivatives demonstrated excellent photothermal therapeutic and imaging performance both in vitro and in vivo. Notably, compound **17** achieved an optimal balance of fluorescence, photothermal, and photodynamic properties, exhibiting a relatively high fluorescence quantum yield (3.0%), PCE (54.9%), and singlet oxygen production (40.76%) (Figure 12b–e). These features identify compound **17** as a particularly promising candidate for cancer diagnosis and synergistic phototherapy.

### 3.5 Oblique‐Type Aggregates

**Figure 13:**
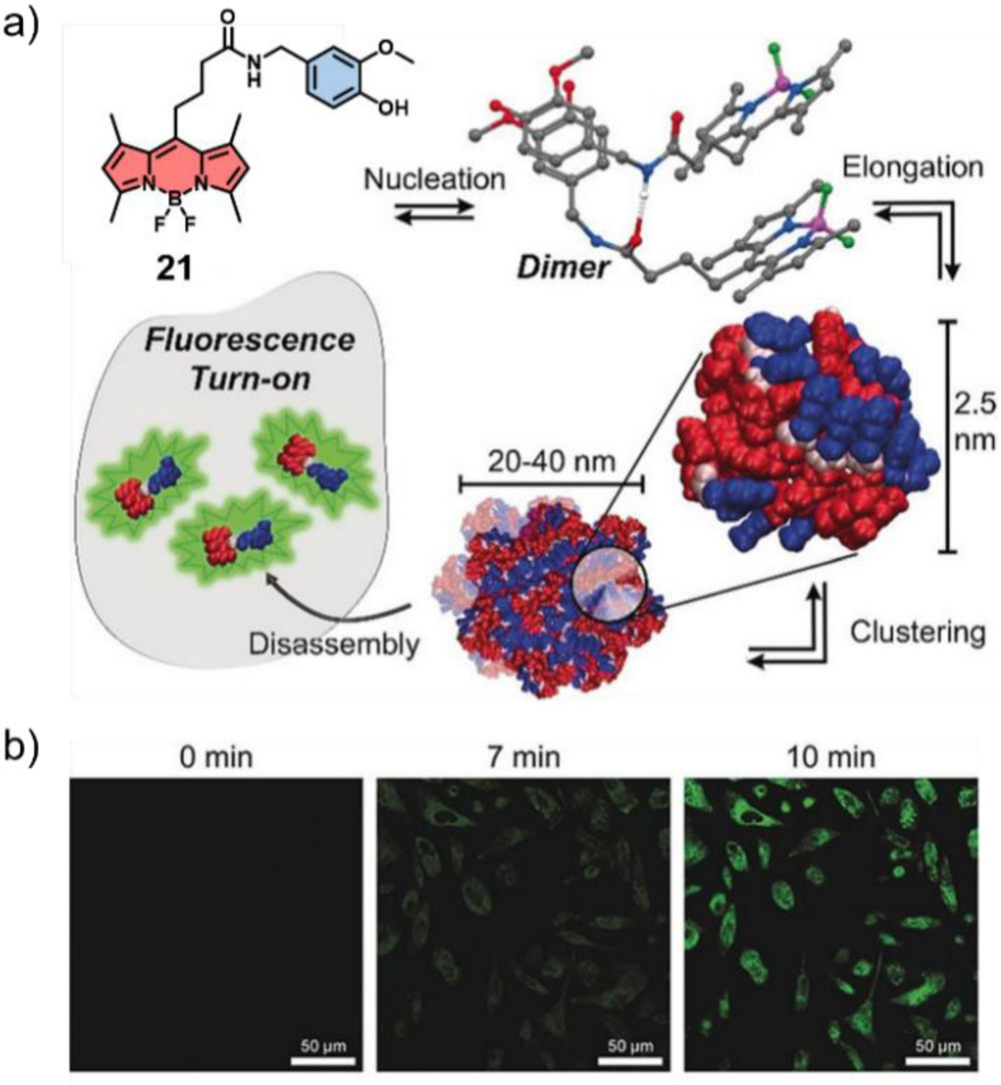
a) Chemical structure of **21**, proposed hierarchical aqueous self‐assembly and subsequent disassembly‐driven emission turn‐on upon cellular uptake. b) Time‐dependent evolution micrographs of PC3 (prostate cancer cells) incubated with **21** assemblies. Reprinted from Ref. 34 Copyright 2018 Wiley‐VCH.

Beyond the well‐studied H‐ and J‐type aggregates, assemblies exhibiting oblique‐type exciton coupling are rare.^[^ ^81^ ^]^ Owing to the geometrical orientation of transition dipole moments, this intermediate packing motif permits both high‐ and low‐energy transitions characteristic of H‐ and J‐type species, respectively, resulting in aggregate spectra with both blue‐ and red‐shifted bands. In this regard, our group shed light on the molecular requirements for this unconventional type of packing and their influence on biomedical applications. In our early example, we designed a BODIPY derivative **21** covalently tethered to the anticancer drug capsaicin (CAP) in order to facilitate the formation of well‐defined aqueous CAP assemblies without the need for nanocarrier encapsulation (Figure 13a).^[^ ^34^ ^]^ The spontaneous self‐assembly of compound **21** in water revealed an oblique arrangement of the chromophores, displaying both red‐ and blue‐shifted absorption maxima. These spectral features are attributed to structural heterogeneity resulting from a broad distribution of chromophore twist angles, as well as from hydrophobic and inter‐cluster hydrogen‐bonding interactions between CAP moieties mediated by water molecules. In the aggregated state, fluorescence emission is markedly quenched.^[^ ^82^ ^]^ However, upon cellular uptake, intracellular green fluorescence corresponding to the monomeric species becomes detectable within 30 s and reaches maximal intensity at approximately 6 min (Figure 13b), indicating a cell‐mediated disassembly of the aggregates into monomers. In vivo studies in PC3 (prostate cancer cells) tumor‐bearing mice showed aggregation‐induced accumulation in the TME, consistent with the EPR effect, where nanoparticles preferentially localize due to enhanced tissue porosity and reduced drainage. This allows the effective dose to be reduced by over two orders of magnitude compared to unmodified CAP while maintaining comparable antitumor activity.

**Figure 14:**
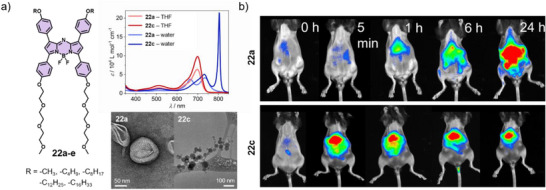
a) Chemical structures of **22a–e** with UV/Vis and TEM analysis of the assemblies. b) In vivo FI of **22a** and **22c** at different timepoints. Reprinted from Ref. 39 Copyright 2025 Wiley‐VCH.

Very recently, we reported a series of amphiphilic aza‐BODIPY derivatives (**22a**–**e**) bearing hydrophobic domains of tunable length that govern nanoparticle size and photophysical properties.^[^ ^39^ ^]^ Compound **22a**, adopting an oblique‐type packing promoted by intermolecular C─H_arom_⋯F interactions, forms non‐emissive nanofibers that coil into concentric layers (∼50 nm). In contrast to in vitro studies, **22a** displayed an in vivo fluorescence turn‐on attributed to the presence of residual monomers that are in equilibrium with the assemblies. On the other hand, derivatives with longer alkyl chains (**22b–e**) self‐assemble into J‐type nanoparticles with adjustable sizes ranging from 100 nm to 1 µm, exhibiting NIR absorption, noticeable emission, and strong photoacoustic signals (Figure 14a). In vitro cytocompatibility assays together with in vivo murine biodistribution studies with ex vivo validation demonstrated high biocompatibility and a size‐dependent organ‐specific biodistribution of the nanostructures (Figure 14b). This work demonstrates the strong potential of molecular design‐driven self‐assembly to precisely tune aggregate size and photophysical properties for effective bimodal in vivo imaging.

### 3.6 (Aza)BODIPY Assemblies Exhibiting Pathway Complexity

Beyond single aggregate systems, increasing attention has been directed toward pathway complexity, in which the same monomer can self‐assemble into multiple supramolecular structures under different kinetic or thermodynamic conditions. The isolation of kinetically trapped and thermodynamically stable aggregates from the same building block offers an additional dimension of structural and functional tunability. Exploiting distinct assembly pathways provides the opportunity to expand the range of accessible imaging modalities, for instance through selective activation or turn‐on responses arising from environment‐dependent aggregate interconversion.

**Figure 15:**
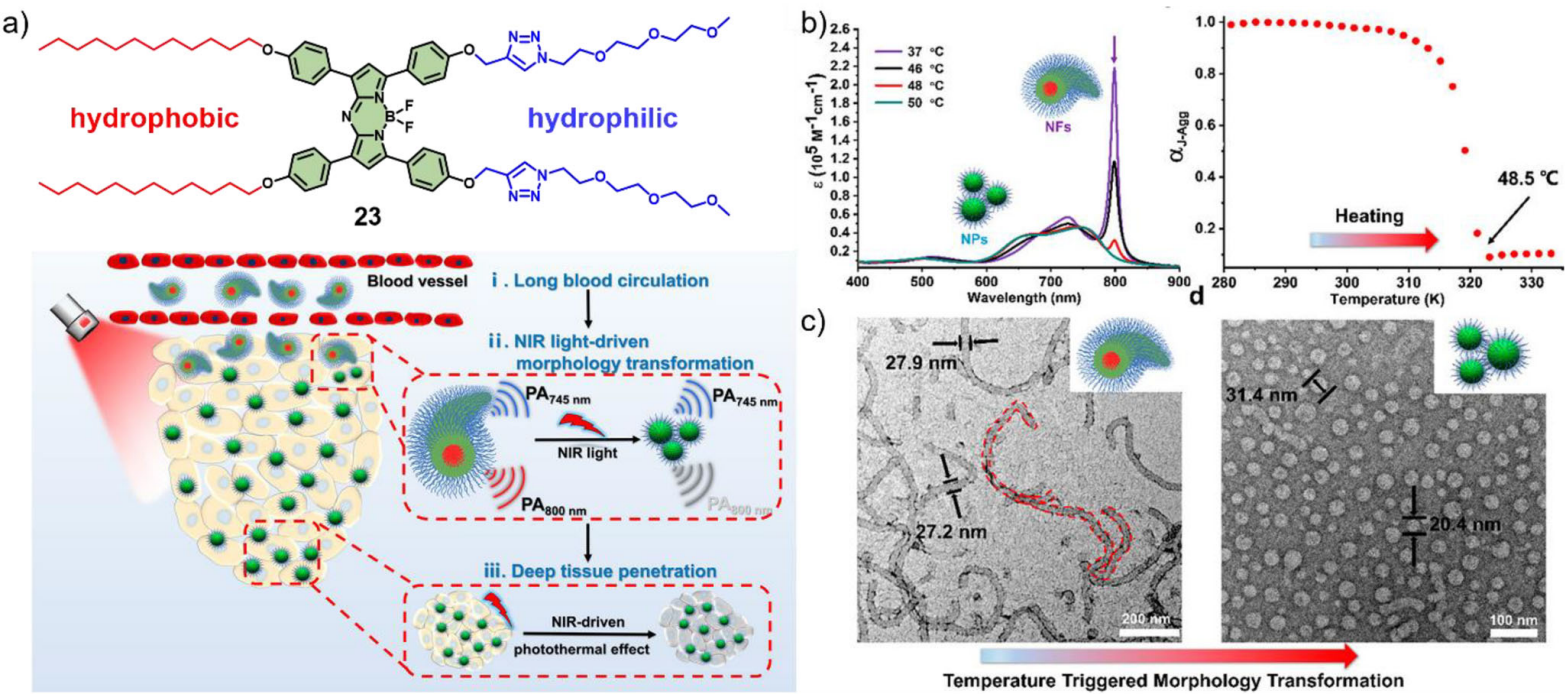
a) Chemical structure of **23** and schematic illustration of the in vivo morphology transformation for enhanced long blood circulation and deep tissue penetration. b) UV/Vis absorption spectra of the transformation of nanofibers (NFs) into nanoparticles (NPs) in PBS upon increasing the temperature and the corresponding plot of the molar fraction monitored at 799 nm as a function of temperature. c) TEM images of NFs at room temperature and of NPs obtained at 50 °C. Reprinted from Ref. 46 Copyright 2020 American Chemical Society.

In an illustrative example, Chen, Qiao, Wang, and co‐workers demonstrated an amphiphilic aza‐BODIPY derivative **23** that undergoes an in situ morphological transition between two well‐defined aggregated states with clearly distinguishable photophysical characteristics.^[^ ^46^ ^]^ Initially, **23** self‐assembles into J‐type worm‐like nanofibers (NFs) with prolonged systemic circulation. Upon increasing the temperature, these NFs undergo a reversible transition into spherical nanoparticles (NPs), which display enhanced penetration into solid tumors (Figure 15a,b). This temperature increase can be achieved locally by NIR irradiation at 655 nm exploiting the intrinsic photothermal effect (∼48 °C) of **23**. The resulting morphological transformation (Figure 15c) improves both tumor penetration and PTT efficacy (PCE of 40.1%). Notably, this transition is accompanied by distinct spectral changes and corresponding shifts in photoacoustic signals, enabling real‐time monitoring via PAI. In vivo, the NFs circulate approximately 7.6 times longer than the NPs, subsequently accumulate at tumor sites, and ultimately transform into NPs under NIR laser exposure, achieving deep tumor penetration and effective tumor inhibition.

**Figure 16:**
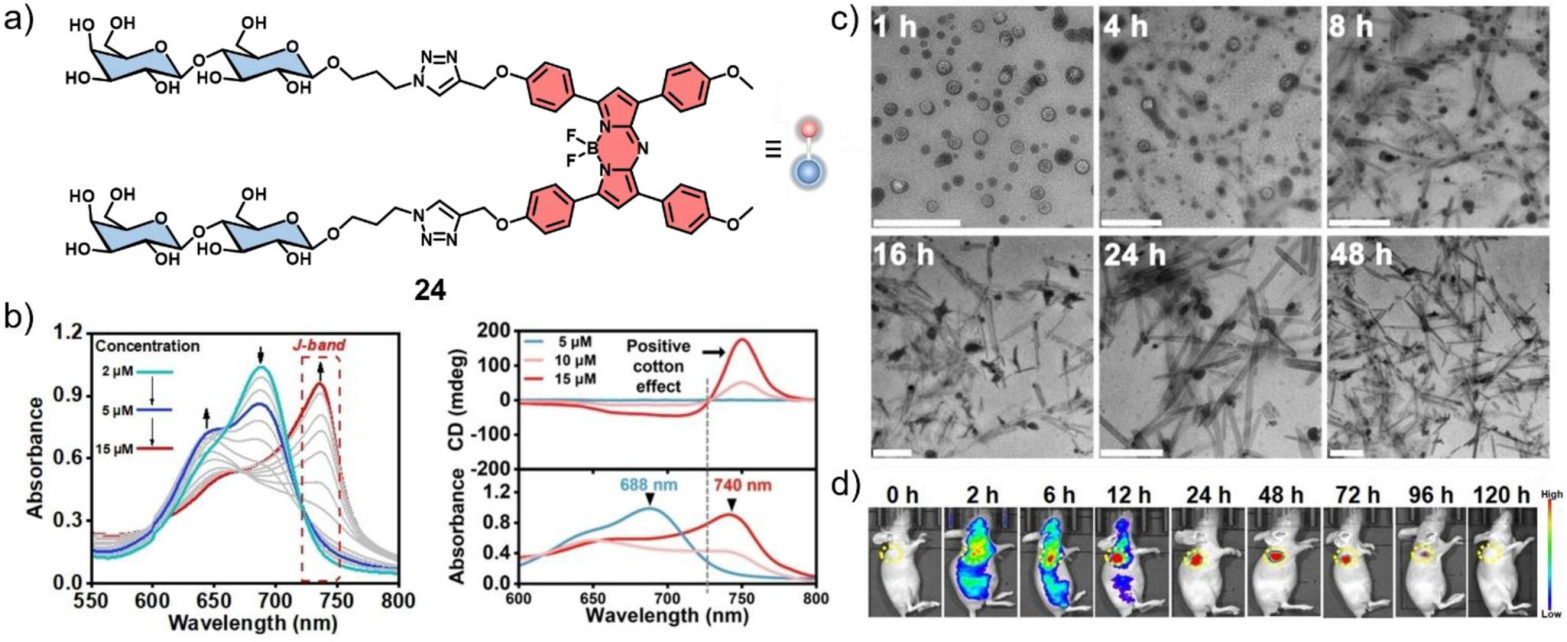
a) Chemical structure of **24**. b) UV/Vis absorption and CD spectra of **24** in water at different concentrations showing two different aggregated species. c) TEM images at different timepoints (scale bar: 500 nm). d) In vivo FI of HepG2 tumor‐bearing mice. Reprinted from Ref. 83 Copyright 2023 Wiley‐VCH.

In a subsequent work, Xing, and co‐workers reported on a lactose‐modified aza‐BODIPY **24** featuring strong biological receptor binding ability and exceptional stability (Figure 16a).^[^ ^83^ ^]^ In aqueous solutions, **24** self‐assembles in a concentration‐dependent manner into J‐type aggregated nanostructures (Figure 16b). Initially, **24** forms ROS‐generating nanovesicles suitable for type I PDT, and subsequently transforms via carbohydrate–carbohydrate interactions into persistent nanofibers that are able to accumulate at cell membranes (Figure 16c). This vesicle‐to‐nanofiber evolution not only enhances type I ROS generation but also disrupts membrane integrity, yielding synergistic cytotoxicity. In vivo FI imaging showed that intravenously administered **24** selectively accumulated at HepG2 tumor sites in BALB/c mice, exhibiting strong and prolonged retention up to 96 h. On the other hand, the fluorescence in major organs diminished after 48 h (Figure 16d), a phenomenon attributed to the nanofiber formation of **24** at the tumor site.

## 4 Discussion

**Scheme 2:**
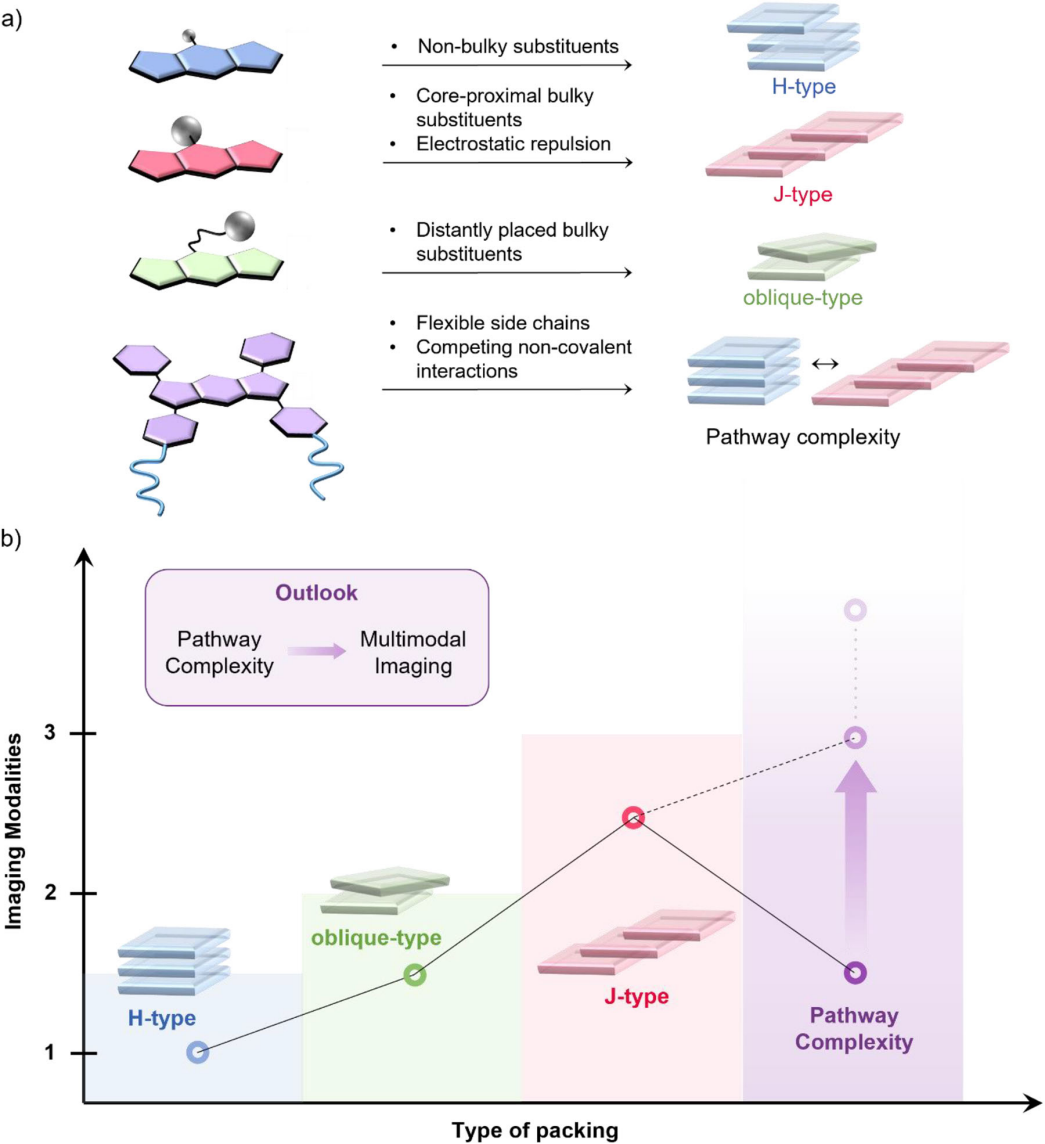
Perspective on a) the relationship between molecular design and packing motif, and b) packing mode and potential number of imaging modalities.

The previous sections have summarized recent advances in carrier‐free (aza)BODIPY‐based self‐assemblies for biomedical imaging; however, a systematic comparison of their structure‐function relationship has not yet been undertaken. To address this gap, this section categorizes the reported CAs according to their packing motifs and corresponding (multi)modal imaging capabilities, aiming to establish direct correlations between molecular design strategies, exciton coupling types, and the resulting photophysical properties (Scheme 2a).

A careful analysis of the literature on self‐assembled (aza)BODIPYs reveals several general trends:

1. Consistent with the behavior of other dye assemblies, small or non‐bulky substituents at the BODIPY core typically promote face‐to‐face stacking, leading to H‐type exciton coupling.
2. The introduction of repulsive interactions―such as steric hindrance from bulky groups or electrostatic repulsion―prevents ideal face‐to‐face stacking, resulting instead in a slipped arrangement characteristic of J‐type aggregates. A common strategy to achieve J‐type aggregation relies on the incorporation of bulky substituents, such as aromatic groups, at the BODIPY *meso*‐position.
3. Although still rare, intermediate oblique‐type packings can be obtained when sufficient spatial separation is maintained between bulky substituents and the chromophoric core. However, disentangling the roles of hydrophobic and solvophobic effects in these assemblies remains challenging, as both factors critically influence the type and strength of intermolecular associations.^[^ ^34^, ^62^, ^84^, ^85^ ^]^

Extrapolating these insights to (aza)BODIPYs is more complex, not only due to the smaller number of reported examples compared to BODIPYs, but also because of their different synthetic accessibility and functionalization patterns.

When the reported CAs are analyzed according to their imaging capabilities in relation to exciton coupling, a clear trend emerges: as the absorption of the aggregated species shifts bathochromically (H‐type → oblique‐type → J‐type), the potential for multimodal imaging increases (Scheme 2b). While H‐type aggregates are generally limited to single‐modality applications, the deliberate induction of J‐type packing can expand functionality toward bi‐ or trimodal imaging.

A particularly promising avenue lies in the design of systems exhibiting pathway complexity, wherein a single molecule can access multiple aggregated states with distinct photophysical and imaging characteristics. Beyond tuning external conditions such as temperature, concentration, or solvent composition, pathway complexity can be promoted through rational molecular design. Effective strategies include:

1. Incorporating multiple competing non‐covalent interaction motifs within a single molecule^[^ ^84^ ^]^ or
2. Introducing flexible linkers—such as triazoles—between the chromophore and the solubilizing chains, which can facilitate structural rearrangements under varying conditions, a strategy that has proven particularly successful for aza‐BODIPY dyes (Scheme 2a).^[^ ^46^, ^50^, ^83^ ^]^

Therefore, it is conceivable that assemblies previously described as forming a single H‐type aggregate with only one imaging modality may in fact possess latent pathway complexity—and thus additional imaging capabilities yet to be uncovered. This highlights that the full potential of pathway complexity in (aza)BODIPY assemblies has yet to be unlocked, partly because prior studies have emphasized biomedical performance over the fundamental elucidation of self‐assembly mechanisms.

## 5 Conclusion and Outlook

This minireview highlights the versatility of (aza)BODIPYs as a powerful platform for designing functional supramolecular systems that bridge molecular engineering and biomedical imaging. Owing to their outstanding photophysical properties, (aza)BODIPY chromophores represent exemplary building blocks for constructing self‐assembled architectures with precise control over dye arrangement and, consequently, optical and imaging characteristics.

Since biomedical imaging outcomes of dye aggregates are governed by the chromophore arrangement within these assemblies, this work advances beyond the current state of the art by deepening the understanding of structure–property–bioimaging relationships and providing guidance for the rational design of multiscale CAs. Particularly promising is the concept of pathway complexity, which enables a single molecule to access multiple aggregated states with distinct photophysical signatures. This concept introduces a dynamic dimension to supramolecular self‐assembly, endowing nanostructures with stimuli‐responsive and multifunctional imaging capabilities.

Our comparative analysis further reveals that systematic manipulation of exciton coupling—from H‐type to oblique‐type to J‐type packing—can be leveraged to fine‐tune photophysical properties and expand imaging functionality. Future research should combine precise molecular design with advanced spectroscopic and structural characterization to elucidate the dynamic self‐assembly pathways of these systems. Such integrated approaches will not only deepen mechanistic insights into supramolecular polymerization but also accelerate the rational development of next‐generation (aza)BODIPY assemblies for sophisticated biomedical imaging applications.

In conclusion, the classification framework presented her links molecular design, exciton coupling during self‐assembly, and imaging function, providing a conceptual foundation for engineering (aza)BODIPY materials with broadened, tunable, and synergistic capabilities for multimodal biomedical imaging.

## Conflict of Interests

The authors declare no conflict of interest.

## Data Availability

Data sharing is not applicable to this article as no new data were created or analyzed in this study.

## References

[1] B. Matarranz , G. Fernández , Chem. Phys. Rev. 2021, 2, 041304, 10.1063/5.0065873.

[2] Z. Chen , Z. Chen , Org. Chem. Front. 2023, 10, 2581–2602, 10.1039/D3QO00148B.

[3] A. Loudet , K. Burgess , Chem. Rev. 2007, 107, 4891–4932. 17924696 10.1021/cr078381n

[4] A. Bessette , G. S. Hanan , Chem. Soc. Rev. 2014, 43, 3342–3405, 10.1039/C3CS60411J. 24577078

[5] P. Kaur , K. Singh , J. Mater. Chem. C 2019, 7, 11361–11405, 10.1039/C9TC03719E.

[6] T. Kowada , H. Maeda , K. Kikuchi , Chem. Soc. Rev. 2015, 44, 4953–4972, 10.1039/C5CS00030K. 25801415

[7] H.‐B. Cheng , X. Cao , S. Zhang , K. Zhang , Y. Cheng , J. Wang , J. Zhao , L. Zhou , X.‐J. Liang , J. Yoon , Adv. Mater. 2023, 35, 2207546. 10.1002/adma.202207546 36398522

[8] J. C. Hsu , Z. Tang , O. E. Eremina , A. M. Sofias , T. Lammers , J. F. Lovell , C. Zavaleta , W. Cai , D. P. Cormode , Nat. Rev. Methods Primers 2023, 3, 30, 10.1038/s43586-023-00211-4. 38130699 PMC10732545

[9] C. A. J. van Winkel , F. R. Pierik , A. H. Brouwers , D. J. A. de Groot , E. G. E. de Vries , M. N. Lub‐de Hooge , Nat. Rev. Clin. Oncol. 2024, 21, 852–866, 10.1038/s41571-024-00946-3. 39327536

[10] Z. Shi , X. Han , W. Hu , H. Bai , B. Peng , L. Ji , Q. Fan , L. Li , W. Huang , Chem. Soc. Rev., 2020, 49, 7533–7567. 32996497 10.1039/d0cs00234h

[11] H. Lu , J. Mack , Y. Yang , Z. Shen , Chem. Soc. Rev. 2014, 43, 4778–4823, 10.1039/C4CS00030G. 24733589

[12] S. Cherumukkil , S. Ghosh , V. K. Praveen , A. Ajayaghosh , Chem. Sci. 2017, 8, 5644–5649, 10.1039/C7SC01696D. 28989602 PMC5621002

[13] G. Fan , L. Yang , Z. Chen , Chem. Sci. Eng. 2014, 8, 405–417.

[14] J. K. G. Karlsson , A. Harriman , J. Phys. Chem. A 2016, 120, 2537–2546, 10.1021/acs.jpca.6b01278. 27046505

[15] S. Cherumukkil , B. Vedhanarayanan , G. Das , V. K. Praveen , A. Ajayaghosh , Bull. Chem. Soc. Jpn. 2018, 91, 100–120, 10.1246/bcsj.20170334.

[16] Y. Zhu , P. Wu , S. Liu , J. Yang , F. Wu , W. Cao , Y. Yang , B. Zheng , H. Xiong , Angew. Chem. Int. Ed. 2023, 62, e202313166, 10.1002/anie.202313166. 37817512

[17] K. Li , X. Duan , Z. Jiang , D. Ding , Y. Chen , G.‐Q. Zhang , Z. Liu , Nat. Commun. 2021, 12, 2376, 10.1038/s41467-021-22686-z. 33888714 PMC8062432

[18] Y. Zhu , P. Wu , S. Liu , J. Yang , F. Wu , W. Cao , Y. Yang , B. Zheng , H. Xiong , Angew. Chem. Int. Ed. 2023, 62, e202313166, 10.1002/anie.202313166. 37817512

[19] Y. Tian , D. Yin , Q. Cheng , H. Dang , C. Teng , L. Yan , J. Mater. Chem. B 2022, 10, 1650–1662. 35195126 10.1039/d1tb02820k

[20] S. Sao , I. Mukherjee , P. De , D. Chaudhuri , Chem. Commun. 2017, 53, 3994–3997, 10.1039/C7CC00554G. 28337495

[21] V. Rosiuk , A. Runser , A. Klymchenko , A. Reisch , Langmuir 2019, 35, 7009–7017, 10.1021/acs.langmuir.9b00721. 31081637

[22] F. An , J. Xin , C. Deng , X. Tan , O. Aras , N. Chen , X. Zhang , R. Ting , J. Mater. Chem. B 2021, 9, 9308–9315, 10.1039/D1TB01883C. 34714318 PMC8616829

[23] A. Albers , S. Kuberasivakumaran , Z. Fernández , C. G. Daniliuc , Y. Li , M. Lee , C. Geyer , E. Hofmann , C. Faber , A. Helfen , C. Grashoff , M. Masthoff , G. Fernández , Angew. Chem. Int. Ed. 2025, e202500144. 10.1002/anie.202500144 40035710

[24] C. Wu , X. Huang , Y. Tang , W. Xiao , L. Sun , J. Shao , X. Dong , Chem. Commun. 2019, 55, 790–793, 10.1039/C8CC07768A. 30569923

[25] E. Krieg , M. M. C. Bastings , P. Besenius , B. Rybtchinski , Chem. Rev. 2016, 116, 2414–2477. 26727633 10.1021/acs.chemrev.5b00369

[26] H. Dang , Y. Tian , Q. Cheng , C. Teng , K. Xie , L. Yan , J. Colloid Interface Sci. 2022, 612, 287–297, 10.1016/j.jcis.2021.12.177. 34995865

[27] G. Seo , Y. Jeong , Y. Kim , ACS Materials Lett 2022, 4, 1214–1226, 10.1021/acsmaterialslett.2c00146.

[28] T. Kim , J. Y. Park , J. Hwang , G. Seo , Y. Kim , Adv. Mater. 2020, 32, 2002405, 10.1002/adma.202002405. 32989841

[29] W. Li , Y. Kim , M. Lee , Nanoscale 2013, 5, 7711, 10.1039/c3nr02574h. 23881254

[30] J. Kalathil , A. Antony , P. Sandhya , P. M. A. Sekhar , S. Christopher , D. Perumal , S. Paul , D. Samanta , M. S. Singh , R. Varghese , Opt. Mater. 2025, 3, 520–529.

[31] M. R. Molla , S. Ghosh , Phys. Chem. Chem. Phys. 2014, 16, 26672–26683, 10.1039/C4CP03791J. 25375094

[32] A. Sikder , S. Ghosh , Chem. Front. 2019, 3, 2602–2616.

[33] R. Dong , Y. Zhou , X. Huang , X. Zhu , Y. Lu , J. Shen , Adv. Mater. 2015, 27, 498–526, 10.1002/adma.201402975. 25393728

[34] T. Zhang , C. Ma , T. Sun , Z. Xie , Coord. Chem. Rev. 2019, 390, 76–85, 10.1016/j.ccr.2019.04.001.

[35] E. Antina , N. Bumagina , Y. Marfin , G. Guseva , L. Nikitina , D. Sbytov , F. Telegin , Molecules 2022, 27, 1396, 10.3390/molecules27041396. 35209191 PMC8877204

[36] A. Sampedro , A. Ramos‐Torres , C. Schwöppe , C. Mück‐Lichtenfeld , I. Helmers , A. Bort , I. Diaz‐Laviada Marturet , G. Fernández , Angew. Chem. Int. Ed. 2018, 57, 17235–17239, 10.1002/anie.201804783. 30324638

[37] D. Perumal , M. Golla , K. S. Pillai , G. Raj , A. Krishna , P. K. R. Varghese , Org. Biomol. Chem. 2021, 19, 2804–2810, 10.1039/D1OB00002K. 33720265

[38] J. Zhou , L. Rao , G. Yu , T. R. Cook , X. Chen , F. Huang , Chem. Soc. Rev. 2021, 50, 2839–2891, 10.1039/D0CS00011F. 33524093

[39] B. Xie , Y.‐F. Ding , M. Shui , L. Yue , C. Gao , I. W. Wyman , R. Wang , Eur. J. Nucl. Med. Mol. Imaging 2022, 49, 1200–1210, 10.1007/s00259-021-05622-7. 34816296

[40] M. Kasha , H. R. Rawls , M. A. El‐Bayoumi , Pure Appl. Chem. 1965, 11, 371–392, 10.1351/pac196511030371.

[41] M. S. Barclay , S. K. Roy , J. S. Huff , O. A. Mass , D. B. Turner , C. K. Wilson , D. L. Kellis , E. A. Terpetschnig , J. Lee , P. H. Davis , B. Yurke , W. B. Knowlton , R. D. Pensack , Commun. Chem. 2021, 4, 19, 10.1038/s42004-021-00456-8. PMC9037907 35474961

[42] M. S. Barclay , C. K. Wilson , S. K. Roy , O. A. Mass , O. M. Obukhova , R. P. Svoiakov , A. L. Tatarets , A. U. Chowdhury , J. S. Huff , D. B. Turner , P. H. Davis , E. A. Terpetschnig , B. Yurke , W. B. Knowlton , J. Lee , R. D. Pensack , ChemPhotoChem 2022, 6, e202200039.

[43] J. Matern , Y. Dorca , L. Sánchez , G. Fernández , Angew. Chem. Int. Ed. 2019, 58, 16730–16740, 10.1002/anie.201905724. PMC6900041 31271244

[44] P. A. Korevaar , T. F. A. de Greef , E. W. Meijer , Chem. Mater. 2014, 26, 576–586, 10.1021/cm4021172.

[45] I. Helmers , G. Ghosh , R. Q. Albuquerque , G. Fernández , Angew. Chem. Int. Ed. 2021, 60, 4368–4376, 10.1002/anie.202012710. PMC7898687 33152151

[46] Z. Chen , Y. Liu , W. Wagner , V. Stepanenko , X. Ren , S. Ogi , F. Würthner , Angew. Chem. 2017, 129, 5823–5827, 10.1002/ange.201701788. 28371081

[47] Y. Chen , X.‐H. Zhang , D.‐B. Cheng , Y. Zhang , Y. Liu , L. Ji , R. Guo , H. Chen , X.‐K. Ren , Z. Chen , Z.‐Y. Qiao , H. Wang , ACS Nano 2020, 14, 3640–3650, 10.1021/acsnano.0c00118. 32119522

[48] H. Ahmad , S. Muhammad , M. Mazhar , A. Farhan , M. S. Iqbal , H. Hiria , C. Yu , Y. Zhang , B. Guo , Coord. Chem. Rev. 2025, 526, 216383, 10.1016/j.ccr.2024.216383.

[49] F. Würthner , C. R. Saha‐Möller , B. Fimmel , S. Ogi , P. Leowanawat , D. Schmidt , Chem. Rev. 2016, 116, 962–1052, 10.1021/acs.chemrev.5b00188. 26270260

[50] M. Zhao , B. Li , Y. Fan , F. Zhang , Adv. Healthcare Mater. 2019, 8, 1801650, 10.1002/adhm.201801650. 31094099

[51] D. Dutta , R. R. Nair , N. Kayastha , S. A. Nair , P. Gogoi , New J. Chem. 2023, 47, 16596–16603, 10.1039/D3NJ01063E.

[52] B.‐K. Liu , J. Zheng , H. Wang , L.‐Y. Niu , Q.‐Z. Yang , Mater. Chem. Front. 2023, 7, 5879–5890, 10.1039/D3QM00753G.

[53] D. Zhang , L.‐Y. Peng , K.‐X. Teng , L.‐Y. Niu , G. Cui , Q.‐Z. Yang , ACS Materials Lett 2023, 5, 180–188, 10.1021/acsmaterialslett.2c00972.

[54] D. Zhang , K.‐X. Teng , L. Zhao , L.‐Y. Niu , Q.‐Z. Yang , Adv. Mater. 2023, 35, 2209789, 10.1002/adma.202209789. 36861334

[55] Y. Zhang , X. Zheng , L. Zhang , Z. Yang , L. Chen , L. Wang , S. Liu , Z. Xie , Org. Biomol. Chem. 2020, 18, 707–714, 10.1039/C9OB02373A. 31907494

[56] D. D. La , S. V. Bhosale , L. A. Jones , S. V. Bhosale , ACS Appl. Mater. Interfaces 2018, 10, 12189–12216, 10.1021/acsami.7b12320. 29043778

[57] W. Zhou , Y.‐C. Liu , G.‐J. Liu , Y. Zhang , G.‐L. Feng , G.‐W. Xing , Angew. Chem. Int. Ed. 2025, 64, e202413350, 10.1002/anie.202413350. 39266462

[58] Y.‐C. Liu , G.‐L. Feng , J.‐L. Jie , W. Zhou , G.‐J. Liu , Y. Zhang , H.‐M. Su , G.‐W. Xing , Adv. Healthcare Mater. 2025, 14, 2404253, 10.1002/adhm.202404253. 40045640

[59] F. Würthner , T. E. Kaiser , C. R. Saha‐Möller , Angew. Chem. Int. Ed. 2011, 50, 3376–3410, 10.1002/anie.201002307. 21442690

[60] X. Hu , C. Zhu , F. Sun , Z. Chen , J. Zou , X. Chen , Z. Yang , Adv. Mater. 2024, 36, 2304848, 10.1002/adma.202304848. 37526997

[61] I. Helmers , M. Niehues , K. K. Kartha , B. J. Ravoo , G. Fernández , Chem. Commun. 2020, 56, 8944–8947, 10.1039/D0CC03603J. 32638746

[62] Y. Tian , D. Yin , L. Yan , Nanomed. Nanobiotechno.l 2022, 15, e1831, 10.1002/wnan.1831. 35817462

[63] Y. Zhang , R. Zhao , J. Liu , H. Kong , K. Zhang , Y.‐N. Zhang , X. Kong , Q. Zhang , Y. Zhao , Biomaterials 2021, 275, 120945, 10.1016/j.biomaterials.2021.120945. 34126410

[64] J. Wang , Z. Jiang , C. Huang , S. Zhao , S. Zhu , R. Liu , H. Zhu , Molecules 2023, 28, 2997. 37049760 10.3390/molecules28072997 PMC10096313

[65] T. Zhang , W. Zhang , M. Zheng , Z. Xie , J. Colloid Interface Sci. 2018, 514, 584–591, 10.1016/j.jcis.2017.12.074. 29294445

[66] R. Englman , J. Jortner , Mol. Phys. 1970, 18, 145–164, 10.1080/00268977000100171.

[67] L. Zhu , W. Wu , Molecules 2024, 29, 371, 10.3390/molecules29020371. 38257284 PMC10819122

[68] J. Zhou , Y. Zhang , G, Y.u , M. R. Crawley , C. R. P. Fulong , A. E. Friedman , S. Sengupta , J. Sun , Q. Li , F. Huang , T. R. Cook , J. Am. Chem. Soc. 2018, 140, 7730–7736, 10.1021/jacs.8b04929. 29787269

[69] Z. Guo , Y. Zou , H. He , J. Rao , S. Ji , X. Cui , H. Ke , Y. Deng , H. Yang , C. Chen , Y. Zhao , H. Chen , Adv. Mater. 2016, 28, 10155–10164, 10.1002/adma.201602738. 27714878

[70] G. Chen , M. Xiong , C. Jiang , Y. Zhao , L. Chen , Y. Ju , J. Jiang , Z. Xu , J. Pan , X. Li , K. Wang , Bioorg. Chem. 2024, 148, 107494, 10.1016/j.bioorg.2024.107494. 38797067

[71] D.‐E. Lee , H. Koo , I.‐C. Sun , J. H. Ryu , K. Kim , I. C. Kwon , Chem. Soc. Rev. 2012, 41, 2656–2672, 10.1039/C2CS15261D. 22189429

[72] M. Masthoff , A. Helfen , J. Claussen , W. Roll , A. Karlas , H. Becker , G. Gabriëls , J. Riess , W. Heindel , M. Schäfers , V. Ntziachristos , M. Eisenblätter , U. Gerth , M. Wildgruber , J. Biophoton. 2018, 11, e201800155, 10.1002/jbio.201800155. 29974645

[73] B. Zhao , L. Liao , Y. Zhu , Z. Hu , F. Wu , J. Lumin. 2023, 263, 120099, 10.1016/j.jlumin.2023.120099.

[74] Z. Zhang , W. Tang , Y. Li , Y. Cao , Y. Shang , ACS Biomater. Sci. Eng. 2021, 7, 4503–4508, 10.1021/acsbiomaterials.1c00597. 34437801

[75] T. Guo , Q. Tang , Y. Guo , H. Qiu , J. Dai , C. Xing , S. Zhuang , G. Huang , ACS Appl. Mater. Interfaces 2021, 13, 306–311, 10.1021/acsami.0c21198. 33382584

[76] J. Weber , P. C. Beard , S. E. Bohndiek , Nat. Methods 2016, 13, 639–650, 10.1038/nmeth.3929. 27467727

[77] C. Li , W. Lin , S. Liu , W. Zhang , Z. Xie , J. Mater. Chem. B 2019, 7, 4655–4660. 31364670 10.1039/c9tb00752k

[78] Z. Guo , H. He , Y. Zhang , J. Rao , T. Yang , T. Li , L. Wang , M. Shi , M. Wang , S. Qiu , X. Song , H. Ke , H. Chen , Adv. Mater. 2021, 33, 2004225, 10.1002/adma.202004225. 33270303

[79] W. Feng , Y. Lv , Z. Chen , F. Wang , Y. Wang , Y. Pei , W. Jin , C. Shi , Y. Wang , Y. Qu , W. Ji , L. Pu , X.‐W. Liu , Z. Pei , Chem. Eng. J. 2021, 417, 129178, 10.1016/j.cej.2021.129178.

[80] M. Jiang , J. Zhang , Y. Li , T. Shi , T. Ma , Y. Sun , H. Qiu , Y. Li , S. Yin , Mater. Chem. Front. 2023, 7, 3668–3679, 10.1039/D3QM00239J.

[81] A. Albers , S. Baumert , C. Daniliuc , G. Fernández , Chemistry Europe 2025, e202500253.

[82] H. Wen , Q. Wu , X. Xiang , T. Sun , Z. Xie , X. Chen , ACS Appl. Mater. Interfaces 2024, 16, 61739–61750, 10.1021/acsami.4c14754. 39473240

[83] Y.‐C. Liu , G.‐J. Liu , W. Zhou , G.‐L. Feng , Q.‐Y. Ma , Y. Zhang , G.‐W. Xing , Angew. Chem. Int. Ed. 2023, 62, e202309786, 10.1002/anie.202309786. 37581954

[84] I. Helmers , B. Shen , K. K. Kartha , R. Q. Albuquerque , M. Lee , G. Fernández , Angew. Chem. 2020, 59, 5675–5682, 10.1002/anie.201911531. 31849157 PMC7154731

[85] L. Yang , Y.‐J. Ji , J.‐F. Yin , Y. Wu , H. Fan , Y. Zhang , G.‐C. Kuang , Soft Matter 2016, 12, 8581–8587, 10.1039/C6SM01796G. 27714381

